# Oral Bioinspired Peroxisome‐Engineered Probiotics for Modulating Gut Microbiota Homeostasis and Alleviating Cardiac Chemotherapy Toxicity

**DOI:** 10.1002/advs.202519344

**Published:** 2026-05-08

**Authors:** Shuyu Wang, Xiaowan Fan, Chao Zhang, Shuai Han, Yike Guo, Yuchen Wang, Wei Wang, Junyue Xing, Ying Liu, Wei Jiang, Xiaoying Chen

**Affiliations:** ^1^ Department of Cardiology Cardiovascular Institute of Zhengzhou University The First Affiliated Hospital of Zhengzhou University Zhengzhou China; ^2^ National Health Commission Key Laboratory of Cardiovascular Regenerative Medicine Central China Subcenter of National Center For Cardiovascular Diseases Henan Cardiovascular Disease Center Fuwai Central‐China Cardiovascular Hospital Central China Fuwai Hospital of Zhengzhou University Zhengzhou China; ^3^ Institute of Cardiovascular Disease Henan Academy of Innovations in Medical Science Zhengzhou Henan China; ^4^ State Key Laboratory of Metabolic Dysregulation & Prevention and Treatment of Esophageal Cancer Tianjian Laboratory of Advanced Biomedical Sciences Academy of Medical Sciences Zhengzhou University Zhengzhou China; ^5^ School of Medicine of Henan University Zhengzhou Henan China; ^6^ Nanozyme Laboratory in Zhongyuan Henan Academy of Innovations in Medical Science Zhengzhou Henan China

**Keywords:** doxorubicin‐induced cardiotoxicity, gut‐heart axis, nanozyme, oral targeted therapy, probiotics

## Abstract

Anthracyclines such as doxorubicin (DOX) are widely used in cancer chemotherapy but their clinical utility is severely limited by cumulative, dose‐dependent, and largely irreversible cardiotoxicity. Mounting evidence suggests that DOX disrupts intestinal barrier integrity and microbial homeostasis, aggravating systemic oxidative stress and accelerating myocardial injury through the gut–heart axis. Probiotics offer a potential strategy to stabilize the intestinal microenvironment, yet their fragile nature and poor survival in the gastrointestinal tract hinder clinical translation. Here, we present an orally administrable bioinspired peroxisome engineered probiotic (BPEP) as a safe and effective therapeutic platform. Ruthenium‐based nanozymes with superoxide dismutase‐like and catalase‐like activities were encapsulated in a lipid shell to form bioinspired peroxisomes (BP) and covalently anchored onto *Escherichia coli Nissle 1917*. The lipid shell enhances probiotic resistance to gastric acid, bile salts, and reactive oxygen species, improving gastrointestinal survival and colonization. Acting as a living carrier, probiotics deliver BPs to the intestinal barrier, where they synergistically scavenge reactive oxygen and nitrogen species, restore tight junction integrity, and remodel microbial communities. In a chronic DOX‐induced cardiotoxicity mouse model, oral administration of BPEP effectively alleviated oxidative stress, preserved intestinal barrier function, stabilized microbial homeostasis, and ultimately improved cardiac function. This work establishes a bioinspired probiotic‐nanozyme hybrid strategy that overcomes the intrinsic limitations of natural probiotics and provides a promising approach for mitigating chemotherapy‐related cardiotoxicity via the gut‐heart axis.

## Graphical abstract

This study developed an oral bioinspired peroxisome‐engineered probiotic (BPEP) by anchoring lipid‐encapsulated ruthenium nanozymes onto *Escherichia coli Nissle 1917*, utilizing probiotics to deliver nanozymes to the intestinal barrier for reactive oxygen species/reactive nitrogen species elimination, tight junction restoration, and gut microbiota remodeling, thereby alleviating doxorubicin‐induced cardiotoxicity via the gut‐heart axis.

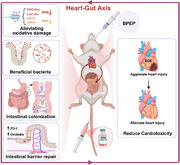

## 1 Introduction

Anthracyclines such as doxorubicin (DOX) have long been the cornerstone of chemotherapy regimens for breast cancer, lymphoma, leukemia, and a wide spectrum of solid tumors due to their potent and broad‐spectrum antitumor activity [1, 2]. However, their clinical utility is severely constrained by cumulative, dose‐dependent, and largely irreversible cardiotoxicity, manifested in excessive oxidative stress, mitochondrial dysfunction, and progressive cardiac impairment [3, 4]. Once established, DOX‐induced cardiotoxicity is difficult to reverse and strongly associated with poor prognosis [5]. Current preventive strategies, including dose adjustment, altered administration schedules, or co‐administration of dexrazoxane, provide only limited benefit and often introduce additional risks, highlighting the urgent need for novel approaches that can effectively mitigate cardiotoxicity without compromising antitumor efficacy.

Recent insights into the gut‐heart axis have highlighted the pivotal role of the intestinal microenvironment in systemic redox regulation and metabolic homeostasis [6, 7, 8, 9]. DOX treatment disrupts intestinal barrier integrity and exacerbates oxidative stress, leading to impaired tight junctions, microbial dysbiosis, and leakage of harmful metabolites into the circulation [10, 11, 12]. Notably, emerging evidence has systematically demonstrated that DOX‐induced intestinal barrier injury precedes the onset of cardiac dysfunction, identifying gut damage as an early upstream event that drives subsequent myocardial injury [12]. This cascade perturbs systemic oxidative balance and accelerates myocardial injury, suggesting that stabilizing the intestinal microenvironment could be a promising strategy to preserve cardiac function during chemotherapy.

Probiotic supplementation offers a potential means of restoring barrier integrity and microbial homeostasis [13, 14, 15]. Probiotics inherently interact with the intestinal mucosa to establish colonization and maintain epithelial homeostasis [16, 17]; however, their survival and long‐term retention in vivo are severely limited by gastric acid, bile salts, and oxidative stress [18, 19]. To overcome these barriers, surface modification strategies such as lipid‐based coatings, carbon‐coated, and carbon quantum dots have been reported to significantly enhance probiotic stability, promote intestinal adhesion, and extend functional persistence within the gastrointestinal tract [20, 21, 22]. Nevertheless, how to integrate protective encapsulation with active redox regulation in situ remains an unmet challenge.

Nanozymes, a class of nanomaterials with intrinsic enzyme‐mimicking activities, provide a promising solution [23]. Unlike natural enzymes, nanozymes exhibit superior stability under harsh physiological conditions and can be engineered with multienzyme cascade activity, such as superoxide dismutase (SOD)‐like and catalase (CAT)‐like activities, enabling efficient elimination of O_2_^·^^−^ and H_2_O_2_ [24, 25]. In addition, nanozymes are also capable of scavenging nitrogen‐containing reactive species, such as nitrogen‐containing reactive species (·ON), thereby providing broader antioxidative defense [26]. Antioxidant nanozymes have already shown therapeutic potential in oxidative stress related disorders by restoring intestinal barrier integrity and remodeling microbial homeostasis [27, 28, 29, 30]. In eukaryotic cells, peroxisomes act as natural antioxidant organelles that integrate multiple enzymes to regulate reactive oxygen species (ROS) metabolism and maintain redox equilibrium [31, 32]. This biological paradigm has inspired the design of bioinspired peroxisome (BP), which aims to replicate the cooperative antioxidative defense of natural peroxisomes through the integration of multienzyme‐like nanozymes.

**SCHEME 1:**
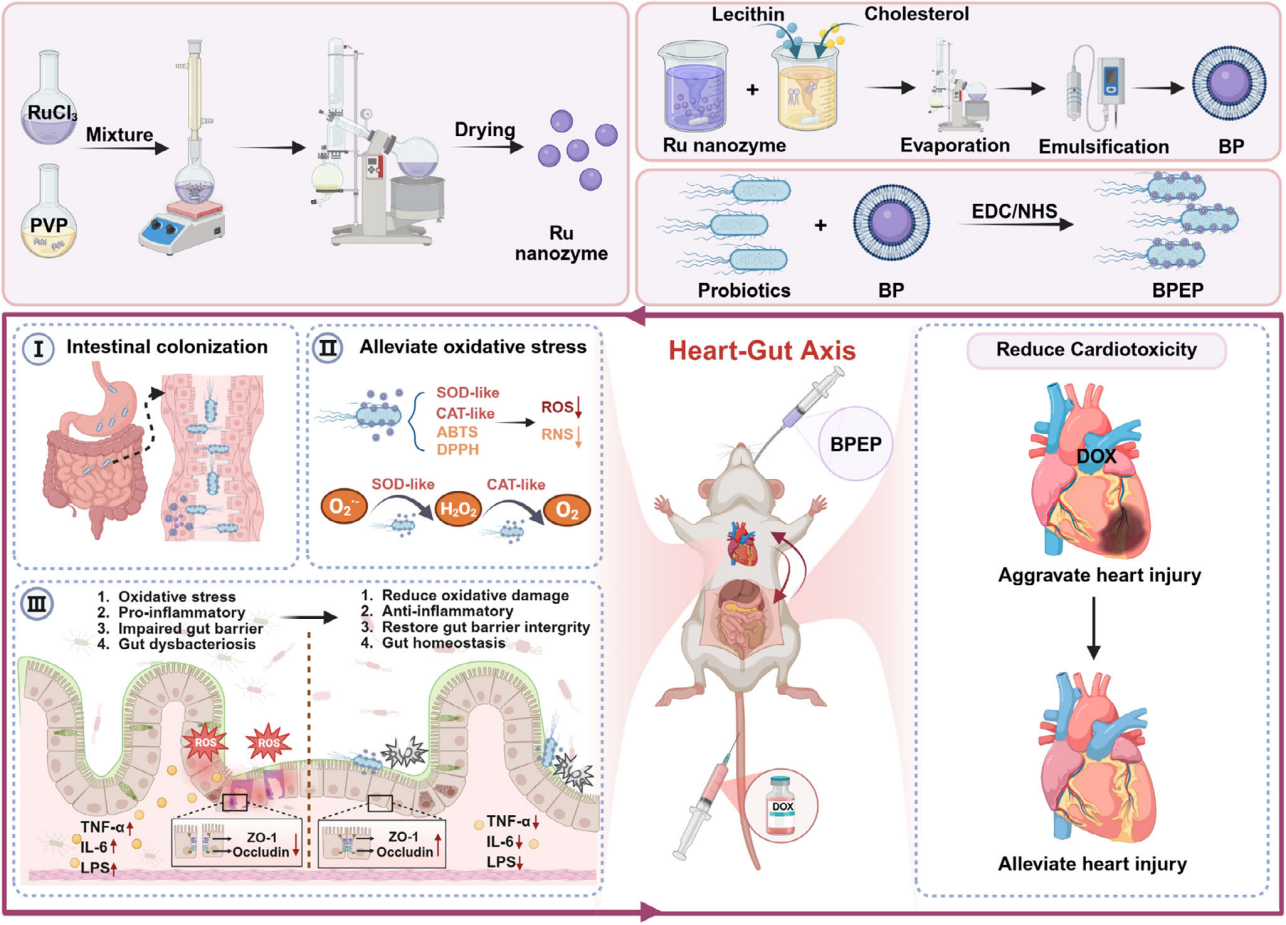
Schematic illustration of the bioinspired peroxisome‐engineered probiotic (BPEP). Created in BioRender. Ru nanozymes with dual SOD‐ and CAT‐like activities are encapsulated in a lipid shell to form BP and covalently anchored onto Probiotics. The lipid layer protects probiotics from gastric acid, bile salts, and ROS, enhancing gastrointestinal survival and colonization. Probiotics deliver BPs to the intestinal barrier, where they synergistically scavenge ROS, repair barrier integrity, and regulate microbial homeostasis, ultimately alleviating DOX‐induced cardiotoxicity via the gut‐heart axis.

In this study, we developed an orally administrable bioinspired peroxisome engineered probiotic (BPEP). Ru nanozymes with dual SOD‐ and CAT‐like activities were synthesized and encapsulated in a lipid shell to form bioinspired peroxisomes (BP), which were covalently anchored onto the surface of *Escherichia coli Nissle 1917* (hereafter referred to as Probiotics). The lipid layer functions as a protective barrier, markedly enhancing probiotic tolerance to gastric acid, bile salts, and ROS, thereby improving gastrointestinal survival and intestinal colonization. Probiotics simultaneously act as a living carrier to deliver BPs directly to the intestinal barrier, where they synergistically eliminate ROS, restore tight junction integrity, and stabilize microbial communities. In a chronic DOX‐induced cardiotoxicity mouse model, BPEP effectively alleviated oxidative stress, preserved barrier function, maintained microbial homeostasis, and ultimately improved cardiac function via the gut‐heart axis (Scheme 1). This work establishes a safe, efficient, and fully oral probiotic‐nanozyme hybrid platform that overcomes the intrinsic limitations of natural probiotics and introduces a bioinspired peroxisome‐based strategy for mitigating chemotherapy‐related cardiotoxicity.

## 2 Results and Discussion

### 2.1 Structural Characterization of BPEP Nanozyme

**FIGURE 1:**
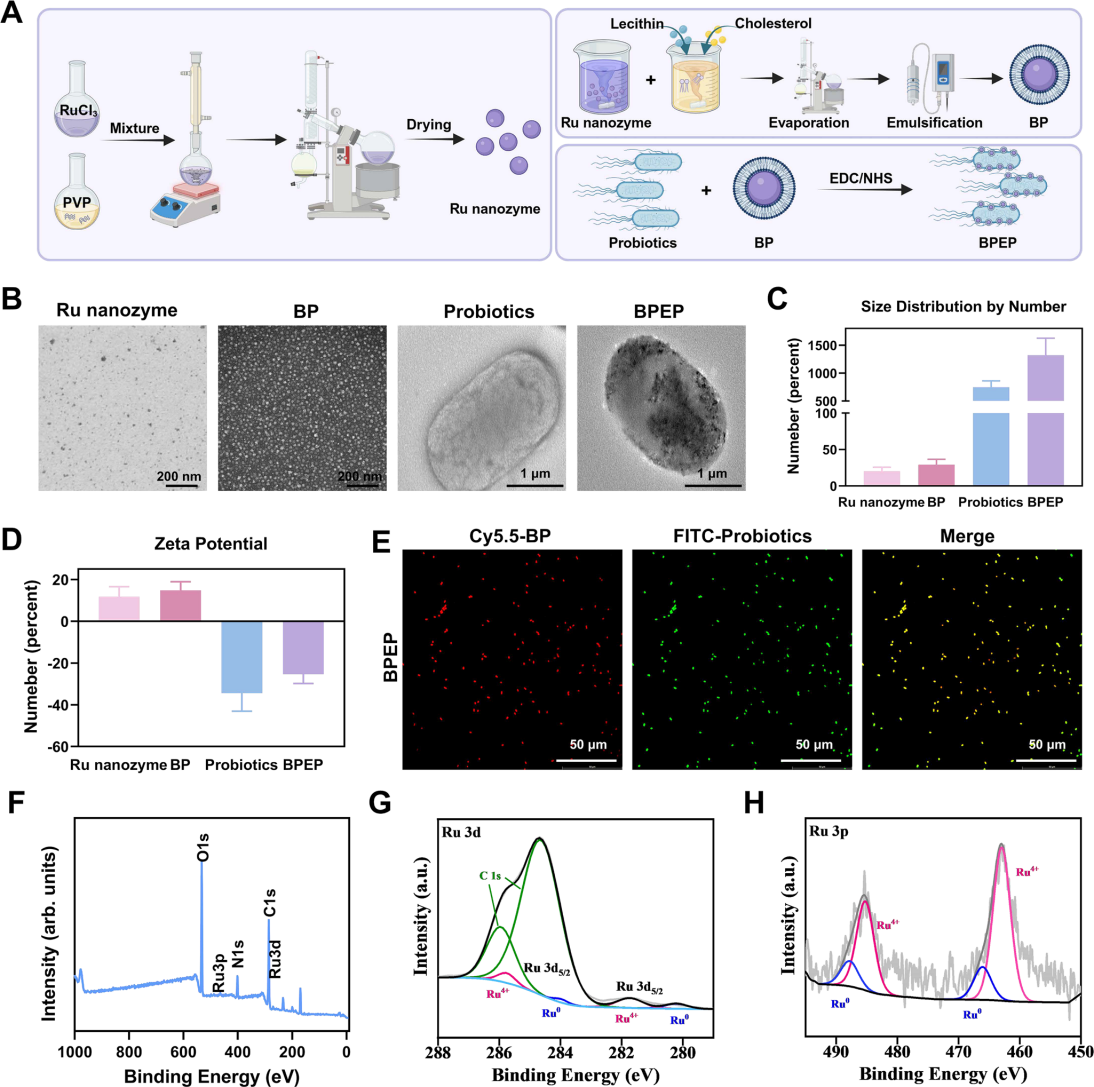
Structural design and physicochemical characterization of BPEP nanozyme. (A) Schematic illustration of BPEP nanozyme synthesis. (B) TEM images of Ru nanozyme, BP, Probiotics, and BPEP nanozymes. (C) Hydrodynamic diameters of Ru nanozyme, BP, Probiotics, and BPEP nanozymes (*n* = 3). (D) Zeta potentials of Ru nanozyme, BP, Probiotics, and BPEP nanozymes (*n* = 3). (E) CLSM images showing the colocalization of Cy5.5‐labeled BP (red) and FITC‐labeled probiotics (green) within BPEP. Scale bar = 50 µm. (F) Elemental composition of BPEP nanozymes determined by XPS. (G) High‐resolution XPS spectra of Ru 3d for BPEP nanozyme. (H) High‐resolution XPS spectra of Ru 3p for BPEP nanozyme.

The BPEP nanozyme was constructed by covalently anchoring lipid‐coated Ru nanozymes onto the surface of *Escherichia coli Nissle 1917*, yielding a bioinspired peroxisome‐engineered probiotic (Figure 1A). Transmission electron microscopy (TEM) revealed that the uncoated Ru nanozymes were uniformly dispersed and spherical, and their morphology and dispersion were well maintained after lipid encapsulation. Compared with the smooth surface of native probiotics, BPEP exhibited a roughened membrane with numerous nanoscale features, and distinct dark BP were clearly visible on the bacterial surface, directly confirming successful anchoring of BP (Figure 1B). Scanning electron microscopy (SEM) further confirmed that BP was successfully coupled to the surface of probiotics (Figure S1).

Dynamic light scattering (DLS) measurements showed a stepwise increase in hydrodynamic size from Ru nanozymes to BP and finally to BPEP, consistent with the progressive assembly and surface modification process (Figure 1C; Figure S2). Zeta potential analysis further indicated a charge reversal upon BP conjugation: BP exhibited a surface potential of 11.89 ± 4.63 mV, whereas BPEP displayed a negative potential of ‐25.40 ± 4.43 mV, attributable to the intrinsic negative surface charge of probiotics (−34.48 ± 8.54 mV), further confirming successful surface attachment of BP (Figure 1D; Figure S3). Confocal laser scanning microscopy (CLSM) demonstrated clear colocalization of Cy5.5‐labeled probiotics (red) and FITC‐labeled BP (green), with well‐overlapped fluorescence signals along the bacterial periphery, thereby providing direct microscopic evidence of covalent surface linkage and uniform distribution of BP on the probiotic carrier (Figure 1E).

High‐resolution x‐ray photoelectron spectroscopy (XPS) revealed a uniform distribution of C, N, O, and Ru elements within BPEP (Figure 1F). Detailed analysis of Ru spectra showed the coexistence of 0 and 4^+^ oxidation states, indicating the stability of Ru nanoclusters (Figure 1G,H). Fourier transform infrared (FTIR) spectroscopy revealed that, after coupling with probiotics, BPEP exhibited new amide I (∼1635 cm^−1^) and amide II (∼1530 cm^−1^) bands, which are attributable to bacterial surface proteins (Figure S4). Taken together, these multi‐level, complementary characterizations confirm that BPEP successfully integrates lipid‐coated Ru nanozymes with a probiotic carrier, forming a structurally stable and functionally tunable hybrid system.

### 2.2 Evaluation of SOD‐ and CAT‐Like Activities of BPEP Nanozyme

**FIGURE 2:**
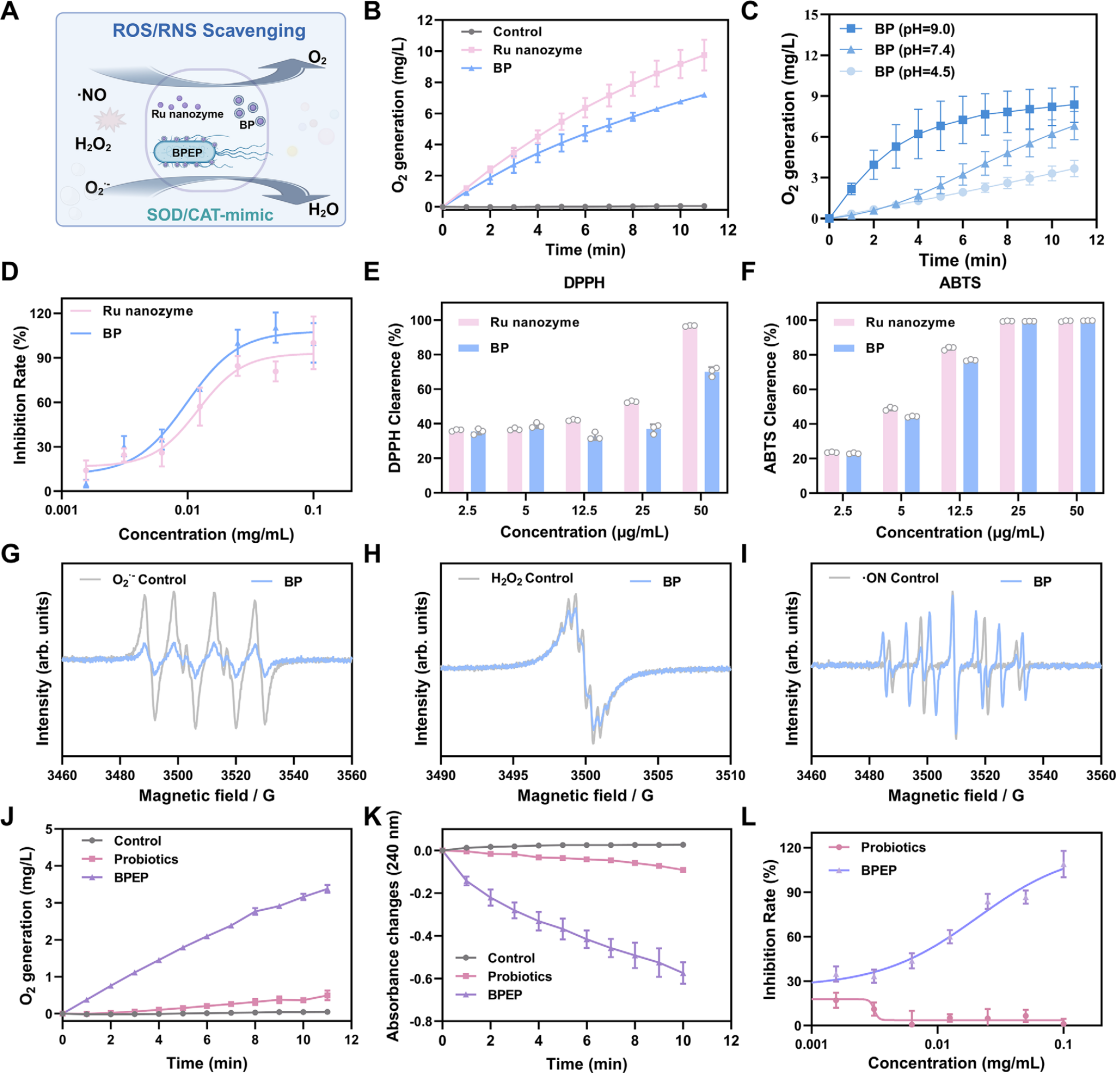
Evaluation of SOD‐ and CAT‐like enzymatic activities of BPEP. (A) Schematic illustration of the nanozyme with SOD‐ and CAT‐like activities for scavenging diverse ROS and RNS. (B) Dissolved oxygen generation of Ru nanozyme and BP in 200 mm H_2_O_2_ solution (*n* = 3). (C) Dissolved oxygen generation of BP in 200 mm H_2_O_2_ solutions at different pH values (*n* = 3). (D) SOD‐like activity evaluation of Ru nanozyme and BP (*n* = 3). (E) Concentration‐dependent DPPH radical scavenging efficiency of Ru nanozyme and BP (*n* = 3). (F) Concentration‐dependent ABTS radical scavenging efficiency of Ru nanozyme and BP (*n* = 3). (G–I) ESR spectra verifying the scavenging of O_2_^·^^−^ (G), H_2_O_2_ (H), and ·ON (I) by BP. (J) Dissolved oxygen generation of Probiotics and BPEP in 200 mM H_2_O_2_ solution (*n* = 3). (K) Time‐dependent H_2_O_2_ decomposition catalyzed by Probiotics and BPEP (*n* = 3). (L) SOD‐like activity evaluation of Probiotics and BPEP (*n* = 3).

To evaluate the ROS‐scavenging capability of the bioinspired peroxisome system, we systematically assessed its SOD‐ and CAT‐like activities, as well as its capacity to eliminate ·ON (Figure 2A). The CAT‐like activity of Ru nanozyme and BP was first compared. In 200 mm H_2_O_2_ solution, BP generated dissolved oxygen at levels comparable to Ru nanozyme within 10 min, indicating that lipid encapsulation did not impair CAT‐like activity. Moreover, BP maintained stable CAT‐like activity across different pH conditions (Figure 2B,C) and over time (Figure S5). SOD‐like activity was evaluated using the cytochrome c reduction assay. BP exhibited superoxide anion scavenging comparable to Ru nanozyme, demonstrating that its efficient SOD‐like activity was preserved after lipid coating (Figure 2D). Additionally, DPPH and ABTS radical scavenging assays demonstrated that both Ru nanozyme and BP possess excellent, concentration‐dependent, and broad‐spectrum antioxidant activity (Figure 2E,F). Electron paramagnetic resonance (EPR) spectroscopy provided direct evidence that BP effectively scavenged O_2_ **^−^**, H_2_O_2_, and ·ON radicals (Figure 2G–I).

Subsequently, we assessed the enzyme‐like activity of BPEP under the same conditions. Both SOD‐ and CAT‐like activities of BPEP were significantly higher than those of unmodified probiotics (Figure 2J–L). Dissolved oxygen generation was markedly enhanced, and H_2_O_2_ decomposition was far more efficient, indicating that the nanozyme retains high catalytic activity when anchored to the bacterial surface. Collectively, these results demonstrate the successful construction of a bioinspired peroxisome‐engineered probiotic system that maintains potent SOD‐CAT cascade activity and broad‐spectrum ROS/RNS scavenging, providing a robust foundation for its application in intestinal oxidative stress regulation and cardioprotection.

### 2.3 In Vitro Biosafety and Antioxidant Efficacy of BP

**FIGURE 3:**
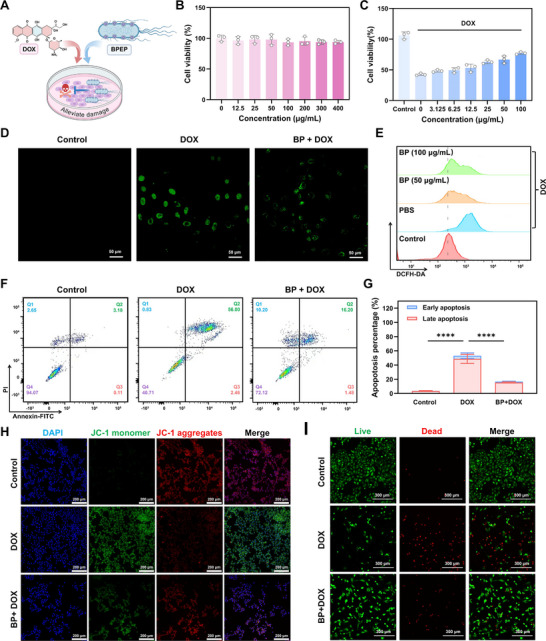
Biomimetic peroxisome scavenges ROS and alleviates DOX‐induced cellular damage. (A) Schematic illustration BP alleviating oxidative stress at the cellular level. (B) Cell viability of H9c2 cells after 24 h exposure to different concentrations of BP (*n* = 3). (C) Protective effects of BP at varying concentrations on H9c2 cells under 500 nm Dox‐induced injury (*n* = 3). (D) Representative fluorescence microscopy images showing intracellular ROS levels in different treatment groups. Scale bar = 50 µm. (E) Flow cytometry analysis of intracellular ROS levels under different treatments. (F) Evaluation of apoptosis levels in H9c2 cells across different treatment groups. (G) Quantitative analysis of apoptosis levels shown in (F) (*n* = 3, one‐way ANOVA with Tukey's multiple comparisons test, ^****^ *p* < 0.0001). (H) Mitochondrial membrane potential staining of H9c2 cells in different treatment groups. Scale bar = 200 µm. (I) Representative live/dead staining images of H9c2 cells under different treatments, with Calcein AM (green) indicating live cells and PI (red) indicating dead cells. Scale bar = 300 µm.

To evaluate the safety and antioxidant function of the BP at the cellular level, we first assessed its intrinsic cytotoxicity toward rat embryonic cardiomyocytes (H9c2) (Figure 3A). CCK‐8 assays showed that H9c2 cells maintained over 90% viability after incubation with BP at concentrations up to 200 µg mL^−1^ for 24 or 48 h, demonstrating excellent biocompatibility and indicating that BP can be safely applied as a potent antioxidant without harming normal cardiomyocytes (Figure 3B; Figure S6).

Next, we evaluated the protective effects of BP in a 500 nM DOX‐induced cardiotoxicity model. BP restored cell viability in a dose‐dependent manner, achieving up to approximately 30% recovery at the highest concentration tested (Figure 3C). A similar protective trend was observed under 200 µm H_2_O_2_‐induced oxidative stress, confirming that BP effectively alleviates oxidative damage across different stress conditions (Figure S7).

To elucidate the underlying mechanism, intracellular ROS levels were measured. DOX treatment markedly increased green oxidative fluorescence in H9c2 cells, whereas BP pretreatment substantially attenuated this signal, indicating efficient ROS scavenging. Flow cytometry and quantitative analysis revealed a leftward shift of the ROS peak, further confirming suppression of free‐radical generation (Figure 3D,E). Consistently, apoptosis analysis showed that the total early and late apoptotic rate decreased from 53.15% ± 5.48% in the DOX group to 16.71% ± 0.84% following BP treatment (Figure 3F,G), indicating that alleviation of oxidative stress directly improved cell survival.

Given that mitochondria are the primary sites of ROS generation and key targets of DOX‐induced damage, mitochondrial membrane potential was assessed using the JC‐1 probe. DOX exposure led to a marked loss of red fluorescence accompanied by an increase in green fluorescence, indicating severe mitochondrial depolarization. In contrast, BP treatment restored red fluorescence and reduced green signals, demonstrating preservation of mitochondrial integrity and energy homeostasis (Figure 3H). Live/Dead staining further visualized these effects: DOX‐treated cultures contained abundant red (dead) cells, whereas BP‐treated cultures were dominated by green (live) cells with significantly fewer dead cells (Figure 3I).

Consistently, in Caco‐2 intestinal epithelial cells, BP exhibited excellent biocompatibility and significantly rescued cell viability following DOX exposure (Figure S8A–C). Moreover, ZO‐1 immunofluorescence staining revealed that BP effectively restored tight junction continuity disrupted by DOX, thereby preserving epithelial barrier integrity (Figure S8D).

Collectively, these in vitro results indicate that BP, through potent SOD‐ and CAT‐like activities, rapidly neutralizes DOX‐induced ROS, mitigates mitochondrial injury, and suppresses apoptosis, providing strong mechanistic support for its subsequent application in protecting against chemotherapy‐related cardiotoxicity.

### 2.4 In Vivo Intestinal Targeting Capability

**FIGURE 4:**
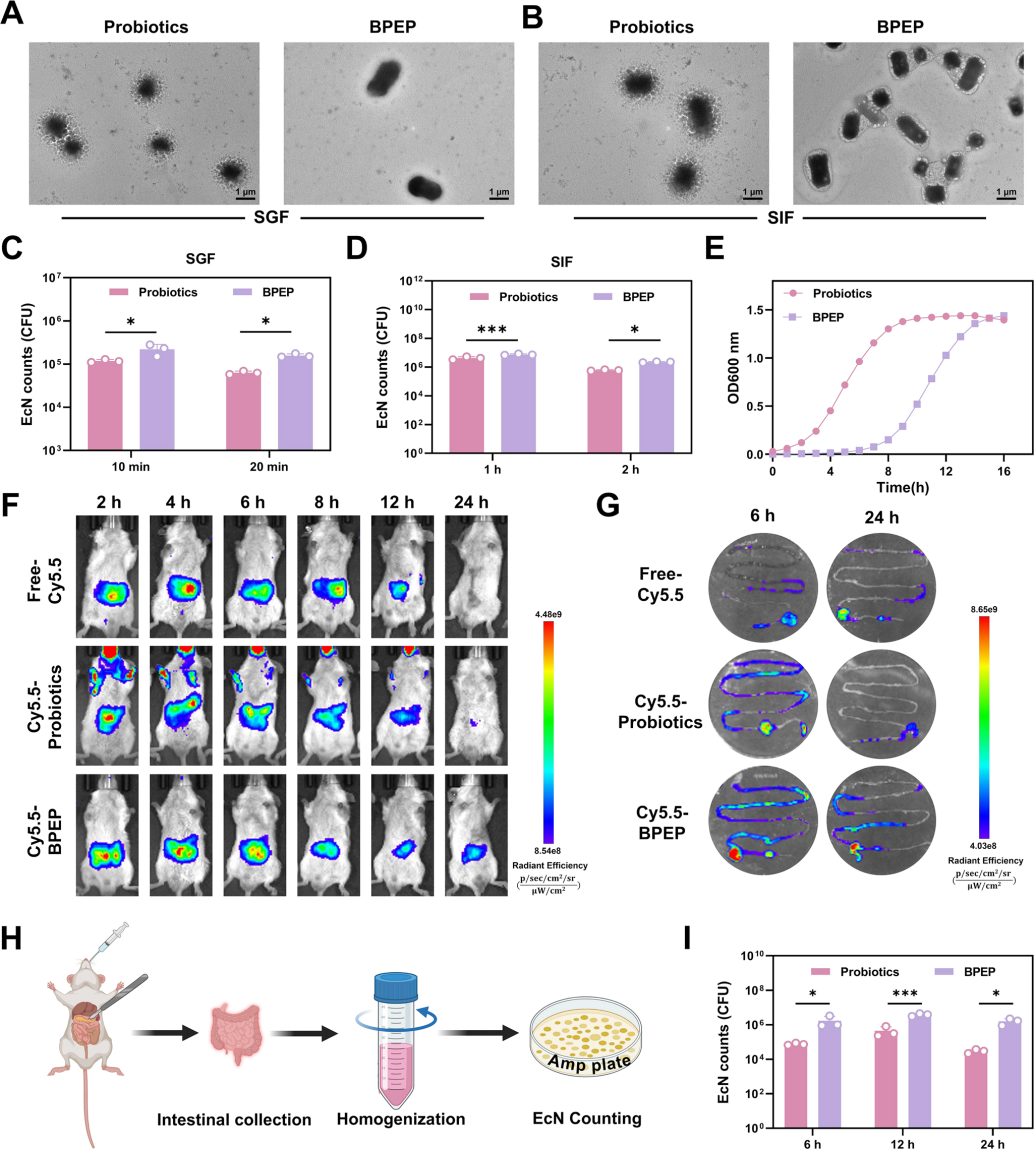
Gastrointestinal stability and in vivo intestinal colonization of BPEP. (A)TEM images of Probiotics and BPEP incubated with SGF for 20 min. Scale bar = 1 µm. (B) TEM images of Probiotics and BPEP incubated with SIF for 2 h. Scale bar = 1 µm. (C,D) Plate colony counts of Probiotics and BPEP after co‐incubation with SGF (C) and SIF (D) (*n* = 3, two‐way ANOVA with Sidak's multiple comparisons test, ^*^ *p* < 0.05, ^***^ *p* < 0.001). (E) Growth curves of Probiotics and BPEP cultured in LB medium at 37 °C (*n* = 3). (F) In vivo fluorescence imaging of mice at 2, 4, 6, 8, 12, and 24 h after oral administration of Free‐Cy5.5, Cy5.5‐Probiotics, or Cy5.5‐BPEP. (G) Ex vivo fluorescence imaging of isolated intestines harvested at 6 and 24 h post‐gavage with Free‐Cy5.5, Cy5.5‐Probiotics, or Cy5.5‐BPEP. (H) Schematic of colony plating of intestinal contents at different time points. (I) Quantification of intestinal bacterial colonies at different time points (*n* = 3, two‐way ANOVA with Sidak's multiple comparisons test, ^*^ *p* < 0.05, ^***^ *p* < 0.001).

To further evaluate whether BPEP can safely traverse the gastrointestinal (GI) barrier and colonize the intestine to exert sustained antioxidant effects, we first performed in vitro simulated GI tests. After 20 min in simulated gastric fluid (SGF), uncoated probiotics exhibited noticeable surface wrinkling and cytoplasmic leakage, whereas BPEP maintained intact rod‐shaped morphology. TEM revealed that the nanozyme layer adhered closely to the bacterial outer membrane without visible damage (Figure 4A). After 2 h of incubation in simulated intestinal fluid (SIF), coated bacteria retained well‐defined edges with no signs of membrane dissolution, indicating that the lipid‐nanozyme shell effectively protects probiotics from gastric acid and bile salts (Figure 4B). Colony‐forming unit (CFU) counts further quantified this protective effect, showing that BPEP survival was significantly higher than that of uncoated bacteria under both SGF and SIF conditions, demonstrating enhanced GI tolerance without compromising membrane permeability (Figure 4C,D). Growth curve analysis in LB medium showed a slightly prolonged lag and stationary phase for BPEP compared with native probiotics, while logarithmic growth rates remained comparable, suggesting that surface modification does not impede bacterial proliferation and supports in situ expansion within the intestine (Figure 4E).

Next, the uptake of BPEP by human colorectal adenocarcinoma Caco‐2 cells was evaluated using hyperspectral microscopy. By establishing a characteristic spectral library of BPEP and matching it with scanned cellular images, we confirmed effective cellular internalization (Figure S9). Biocompatibility was further confirmed by hemolysis assays at different concentrations, demonstrating good hemocompatibility (Figure S10). In addition, hematoxylin and eosin (H&E) staining of major organs after 30 consecutive days of oral administration revealed no pathological abnormalities, confirming the in vivo safety of BPEP at the administered dose (Figure S11). To evaluate BPEP pharmacokinetics and biodistribution, ruthenium (Ru) levels in blood, feces, and major organs were measured by ICP‐MS at 6–72 h post‐administration. Ru concentrations in blood and feces gradually declined over time. By 72 h, Ru was nearly completely cleared from all major organs (Figure S12). These findings indicate the effective clearance and minimal long‐term retention of nanoenzymes, supporting their biological safety.

In vivo studies in mice further validated intestinal targeting and colonization. Compared with Free‐Cy5.5 and Cy5.5‐Probiotics, Cy5.5‐BPEP exhibited enhanced and more persistent intestinal fluorescence, which remained pronounced at 24 h post‐gavage. *Ex vivo* imaging of intestinal segments showed markedly higher fluorescence intensity in the BPEP group, confirming enhanced gut retention (Figure 4F,G; Figure S13). CFU enumeration of intestinal contents demonstrated that BPEP maintained higher viable bacterial counts at 6, 12, and 24 h compared with uncoated probiotics (Figure 4H,I). This enhanced bacterial viability was further confirmed by CCK‐8 assays performed on intestinal contents 24 h after oral administration, which showed significantly higher bacterial activity in the BPEP group, indicating an increased load of viable bacteria (Figure S14). Collectively, these results demonstrate that BPEP prolongs intestinal residence and enhances bacterial survival, supporting its potential for sustained local activity.

Collectively, these results indicate that the nanozyme shell not only preserves the physiological activity of probiotics but also, through a “biological armor” strategy, significantly enhances gastrointestinal stability and intestinal colonization, providing a robust foundation for the sustained in vivo intervention of chemotherapy‐induced cardiotoxicity.

### 2.5 BPEP Alleviates DOX‐Induced Cardiotoxicity Without Compromising its Antitumor Efficacy

**FIGURE 5:**
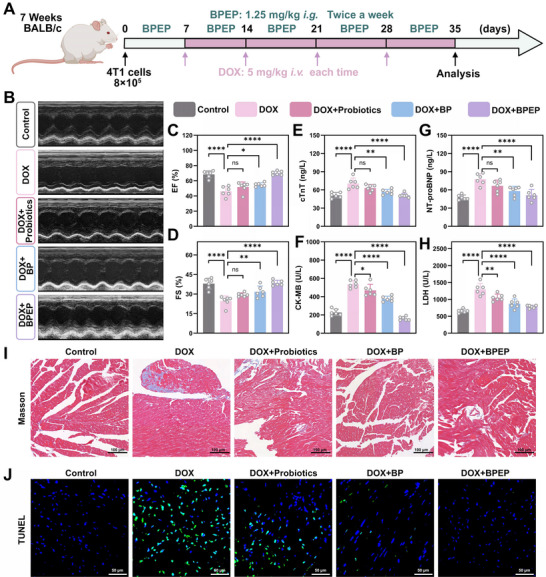
Therapeutic effects of BPEP nanozyme in a Dox‐induced chronic cardiac injury mouse model. (A) Schematic illustration of Dox‐induced chronic cardiac injury model and treatment strategy. (B) Representative echocardiography images of mouse hearts from different treatment groups at the end of treatment. (C,D) Ejection fraction (EF%) (C) and left ventricular fractional shortening (FS%) (D) of mouse hearts in each group (*n* = 6, one‐way ANOVA with Dunnett's multiple comparisons test, ^*^ *p* < 0.05, ^**^ *p* < 0.01, ^****^ *p* < 0.0001). (E–H) Plasma levels of cardiac injury biomarkers in different treatment groups: cTnT (E), CK‐MB (F), NT‐ProBNP (G), and LDH (H) (*n* = 6, one‐way ANOVA with Dunnett's multiple comparisons test, ^*^ *p* < 0.05, ^**^ *p* < 0.01, ^***^ *p* < 0.0001). (I) Masson's trichrome staining of heart tissues from different treatment groups. Scale bar = 100 µm. (J) TUNEL staining of heart tissues in different treatment groups. Scale bar = 50 µm.

To evaluate the protective effect of BPEP against DOX‐induced chronic cardiotoxicity, a 5‐week murine model was established. Chronic cardiac injury was induced via tail‐vein injection of DOX (cumulative dose 20 mg kg^−1^), and oral administration of BPEP commenced one week prior to DOX treatment and continued throughout the experimental period (Figure 5A).

Echocardiographic assessment demonstrated that DOX treatment caused left ventricular wall thinning and impaired systolic function. In contrast, BPEP intervention partially restored cardiac function, with ejection fraction (EF%) and fractional shortening (FS%) improved toward control levels (Figure 5B–D). Serum biomarkers of myocardial injury including cardiac troponin T (cTnT), N‐terminal pro‐brain natriuretic peptide (NT‐proBNP), creatine kinase myocardial band (CK‐MB), and lactate dehydrogenase (LDH) were significantly elevated in BPEP treatment markedly reduced these markers, outperforming either Probiotics or BP alone (Figure 5E–H).

Histological analyses further corroborated the protective effects of BPEP. Masson's trichrome staining revealed extensive myocardial collagen deposition in DOX‐treated mice, whereas BPEP substantially attenuated fibrosis (Figure 5I). TUNEL staining indicated that BPEP reduced DOX‐induced cardiomyocyte apoptosis, providing superior anti‐apoptotic protection compared with other treatment groups (Figure 5J). To directly evaluate oxidative stress in the heart, we quantified lipid peroxidation using 4‐HNE immunohistochemistry and ROS levels via DHE fluorescence staining. BPEP treatment significantly reduced both markers (Figure S15), indicating that the intervention effectively alleviates myocardial oxidative damage.

Considering the critical antitumor role of DOX, we further examined whether BPEP interferes with its cytotoxic efficacy. In 4T1 tumor‐bearing mice, DOX monotherapy effectively suppressed tumor growth, while co‐administration with BPEP produced comparable tumor volumes and terminal tumor weights, with no significant differences in body weight among groups (Figure S16). Individual tumor growth curves similarly confirmed that BPEP did not compromise DOX antitumor activity (Figure S16E–I).

In summary, BPEP effectively alleviates DOX‐induced cardiotoxicity while preserving its antitumor efficacy, achieving “toxicity reduction without efficacy loss” and providing solid preclinical support for its potential translational application.

### 2.6 BPEP Repairs Intestinal Barrier and Microbial Homeostasis

**FIGURE 6:**
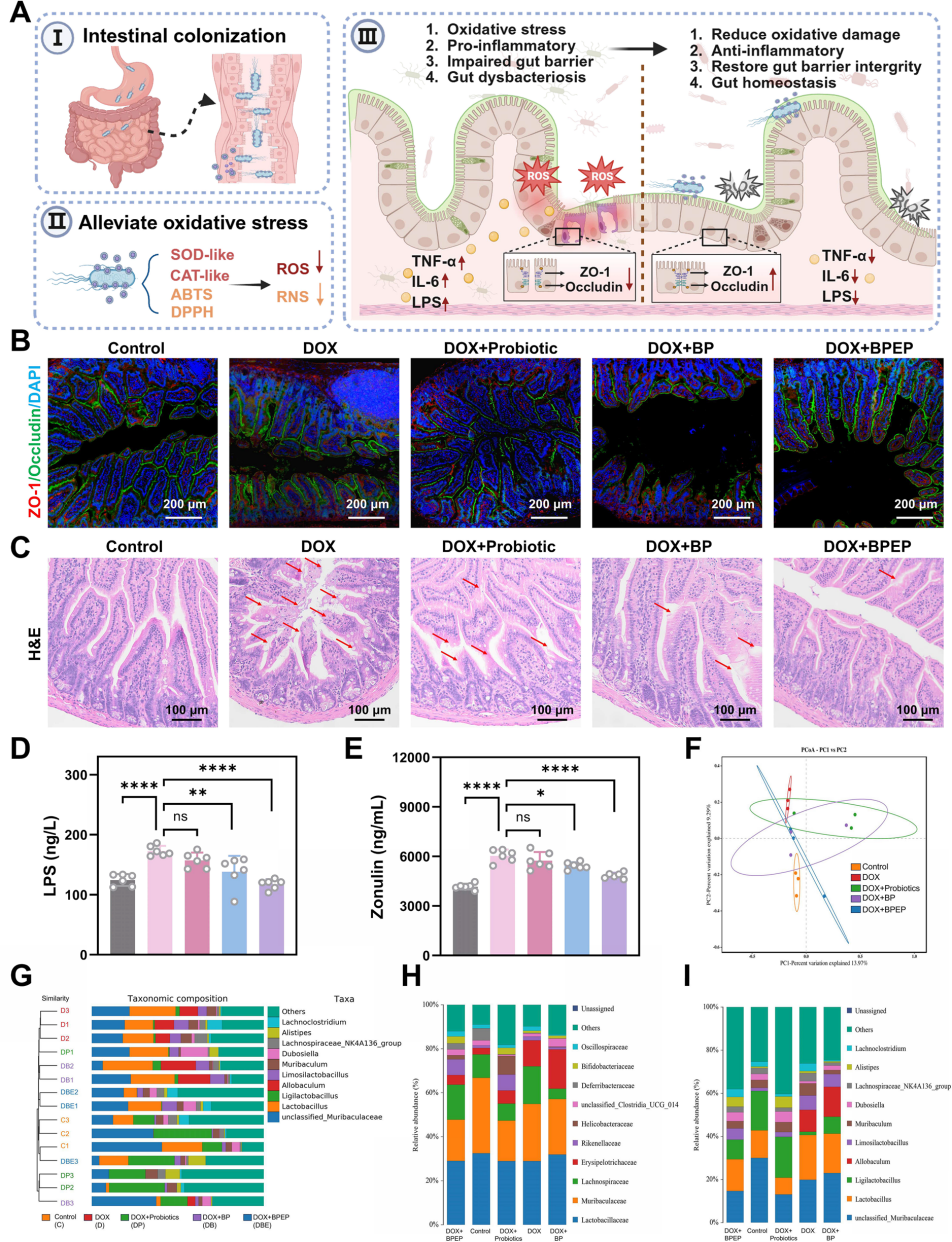
BPEP nanozyme promotes intestinal barrier repair and modulates gut microbiota. (A) Schematic illustration of BPEP nanozyme facilitating intestinal barrier restoration and regulating gut microbiota. (B) Immunofluorescence staining of ZO‐1 and Occludin in intestinal sections from mice across different treatment groups. Scale bar = 200 µm. (C) H&E staining of ileum sections from mice in different treatment groups. Scale bar = 100 µm. (D, E) Plasma levels of LPS (D) and Zonulin (E) in different treatment groups (n = 6, one‐way ANOVA with Dunnett's multiple comparisons test, ^*^ *p* < 0.05, ^**^ *p* < 0.01, ^****^ *p* < 0.0001). (F–I) Gut microbiota analysis across different groups: PCoA analysis (F), UPGMA clustering (G), species composition at the family level (H), and genus level (I).

Having confirmed that BPEP alleviates chemotherapy‐induced cardiotoxicity via the “gut‐heart axis”, we further evaluated its capacity to repair DOX‐disrupted intestinal barriers and restore microbial homeostasis (Figure 6A). Immunofluorescence analysis revealed that in DOX‐treated mice, the tight junction protein ZO‐1 displayed a discontinuous distribution with markedly reduced Occludin expression, accompanied by evident inflammatory cell infiltration, indicating severe tight junction disruption and barrier damage. Following BPEP intervention, ZO‐1 staining along the apical epithelium regained continuous linear patterns, and Occludin expression was substantially restored. While the BP group showed slight improvement compared with the DOX group, likely due to its antioxidant effect, its short intestinal retention and rapid metabolism limited the recovery. In contrast, BPEP exhibited prolonged in vivo retention, leading to superior restoration of tight junction integrity (Figure 6B). This prolonged retention was confirmed by ICP‐MS analysis, which showed significantly higher Ru content in the intestine at 6, 12, and 24 h after BPEP administration compared with BP, demonstrating enhanced nanozyme residence and bioavailability in situ (Figure S17). Consistently, H&E staining demonstrated that the BPEP group preserved intact crypt structures, with mucosal morphology closely resembling that of the control group (Figure 6C).

Furthermore, BPEP effectively attenuated DOX‐induced elevations in circulating markers of intestinal permeability, with serum lipopolysaccharide (LPS) levels decreasing from 172.95 ± 8.55 to 117.48 ± 8.44 mg L^−1^, and zonulin levels declining from 6034.47 ± 375.69 to 4835.33 ± 181.36 mg L^−1^, outperforming other interventions (Figure 6D,E). Correspondingly, systemic inflammatory mediators, including TNF‐α and IL‐6, were partially reduced following BPEP treatment (Figure S18A). Given that the production of these cytokines is regulated by nuclear factor‐κB (NF‐κB), we next examined NF‐κB mRNA expression in cardiac tissue (Figure S18B). Notably, BPEP administration significantly suppressed cardiac NF‐κB expression compared with the DOX group, indicating that BPEP‐mediated restoration of intestinal barrier integrity and attenuation of systemic inflammation translate into downregulation of pro‐inflammatory signaling within the myocardium.

The gut microbiota plays a pivotal role in cardiovascular health. To assess the regulatory effects of BPEP on microbial composition, 16S rRNA high‐throughput sequencing was performed. Principal coordinate analysis (PCoA) revealed distinct microbial differences among the five groups (Figure 6F), with the BPEP group clustering most closely with the control group, suggesting minimal deviation in species diversity. UPGMA analysis further supported this observation: shorter branch lengths indicated higher similarity between the BPEP and control groups, whereas DOX‐treated mice exhibited pronounced compositional divergence (Figure 6G). At both the family and genus levels, DOX treatment caused marked dysbiosis (Figure 6H,I). Specifically, *Erysipelotrichaceae*, a family within Firmicutes known to activate Th17 differentiation and exacerbate inflammation [33], was significantly enriched following DOX exposure. Conversely, anti‐inflammatory *Lactobacillus*‐related genera, such as *Ligilactobacillus* [34], were depleted by DOX but partially restored by BPEP treatment. Oral administration of BPEP reversed these dysbiotic trends, increasing the abundance of anti‐inflammatory *Ligilactobacillus* and restoring microbial composition toward near‐normal levels.

To determine whether microbiota remodeling contributes to BPEP‐mediated cardioprotection, fecal microbiota transplantation (FMT) was performed (Figure S19A). ABX pretreatment effectively reduced endogenous microbiota, as evidenced by a marked decrease in fecal 16S rDNA copy numbers (Figure S19B). Following transplantation and subsequent DOX challenge, recipients of DOX+BPEP‐derived microbiota showed partial improvement in cardiac function, reflected by moderately restored EF% and FS% (Figure S19C–E), accompanied by reduced serum LDH and CK‐MB levels (Figure S19F) and alleviated myocardial fibrosis in Masson's staining (Figure S19G), compared with recipients of DOX microbiota. In contrast, transplantation of DOX microbiota did not noticeably ameliorate cardiac injury. These results suggest that BPEP‐reshaped microbiota contribute, at least in part, to the observed cardioprotective effects under DOX stress.

Collectively, these results demonstrate that BPEP restores intestinal homeostasis through multiple interconnected mechanisms. By virtue of its prolonged intestinal retention, BPEP effectively repairs tight junction integrity, reduces intestinal permeability, and limits systemic leakage of pro‐inflammatory factors, thereby attenuating downstream inflammatory signaling within the myocardium. Concurrently, BPEP counteracts DOX‐induced gut dysbiosis by enriching anti‐inflammatory taxa such as Ligilactobacillus while suppressing pro‐inflammatory lineages including Erysipelotrichaceae. Critically, FMT experiments provide functional evidence that the BPEP‐reshaped microbial community itself confers partial cardioprotection, as transfer of this microbiota alone moderately improves cardiac function and reduces myocardial injury under DOX challenge. Taken together, these findings suggest that BPEP‐mediated intestinal barrier restoration and microbiota remodeling act in concert to alleviate systemic inflammation and subsequent cardiac injury, representing a multifaceted mechanism underlying its therapeutic efficacy against DOX‐induced cardiotoxicity via the gut–heart axis.

## 3 Conclusion

In conclusion, this study demonstrates that BPEP provides an effective strategy for mitigating DOX‐induced chronic cardiotoxicity without compromising its antitumor efficacy. BPEP was rationally designed through the integration of Ru‐based nanozymes possessing dual SOD‐ and CAT‐like activities into a protective lipid shell, followed by covalent conjugation to the probiotic *Escherichia coli Nissle 1917*. This architecture confers exceptional gastrointestinal resilience, promotes sustained intestinal colonization, and facilitates efficient in situ enzyme‐like activity, thereby enabling targeted and persistent antioxidant protection within the gut microenvironment.

Mechanistic investigations reveal that BPEP functions through multiple coordinated pathways: it performs broad‐spectrum scavenging of reactive oxygen and nitrogen species, attenuates intestinal oxidative injury, reinforces epithelial tight junction integrity, reduces gut barrier hyperpermeability, and reprograms the gut microbiota toward a homeostatic and anti‐inflammatory profile. Notably, FMT experiments provide direct causal evidence that BPEP‐reshaped bacterial communities contribute, at least in part, to the observed cardioprotection. These local gastrointestinal improvements subsequently translate into systemic cardioprotection via the gut‐heart axis, ultimately achieving “detoxification without efficacy loss.” These results underscore the significant potential of microbiota‐directed interventions in preventing chemotherapy‐induced distal organ damage.

Beyond its immediate application, this work introduces a generalizable platform for bioinspired peroxisome‐mimetic probiotics, wherein integrated nanozyme activities enable synchronized redox homeostasis and microbial remodeling. The biomimetic design provides a compelling preclinical proof‐of‐concept for BPEP as a safe, efficient oral nanozyme, with potential applicability to other oxidative stress‐associated disorders. We acknowledge that the current microbiota analysis focused on bacterial communities via 16S rRNA sequencing; future studies incorporating multi‐kingdom profiling (e.g., mycobiome and virome) will further elucidate gut ecosystem dynamics and their contribution to organ protection. Collectively, these findings establish a conceptual and technical framework for designing next‐generation probiotic hybrids that functionally connect gut health with systemic physiology, advancing precision supportive therapies in oncology and beyond.

## 4 Experimental Section

### 4.1 Materials

Polyvinylpyrrolidone K30 (PVP K30) was obtained from Bide Pharmaceutical (China). Ruthenium (III) chloride hydrate (RuCl_3_·xH_2_O) was purchased from Aladdin Biochemical (China). Anhydrous methanol and anhydrous ethanol were obtained from Tianli Chemical Reagent Co., Ltd. (Tianjin, China). 2,2′‐Azino‐bis(3‐ethylbenzothiazoline‐6‐sulfonic acid) (ABTS) and 1,1‐diphenyl‐2‐picrylhydrazyl (DPPH) were purchased from Tokyo Chemical Industry (TCI, Japan). Hydrogen peroxide (30% H_2_O_2_) and sodium hydroxide (NaOH) were obtained from McLean Biochemical (China). Cytochrome C (Cyt C) was purchased from Adamas (China). Xanthine oxidase (XO) and xanthine were obtained from Sigma–Aldrich (Shanghai) Co., Ltd. (China). The nanoparticle size and zeta potential were measured using a Malvern Zetasizer Nano ZS90 (UK). Transmission electron microscopy (TEM) images were acquired on an FEI Tecnai 12 TWIN (USA). UV–vis absorbance spectra were recorded using a spectrophotometer from Shanghai Yuanxi Instrument Co., Ltd. (China). Dissolved oxygen measurements were performed using a dissolved oxygen meter from Shanghai Yuanxi Instrument Co., Ltd. (China). Vacuum freeze‐drying was performed using a lyophilizer from Ningbo Xinzhi Biotechnology Co., Ltd. (China).

### 4.2 Synthesis and Preparation of Biomimetic Peroxisome (BP)

Polyvinylpyrrolidone (PVP, 133 mg, 30 kDa) was dissolved in 180 mL of anhydrous methanol. Subsequently, 20 mL of an aqueous RuCl_3_ solution (1 mg/mL) was added to the above solution. The mixture was transferred into a round‐bottom flask and refluxed at 70°C with stirring for 3 h. The reaction solution gradually turned black, indicating the formation of Ru nanoclusters. After completion, the solvent was removed by rotary evaporation, and the crude product was collected in a centrifuge tube. An equal volume mixture of chloroform and n‐hexane was added, and the product was washed multiple times until neutral. The resulting Ru nanozyme was then dried under vacuum. For lipid encapsulation, 4 mg of DSPE‐PEG‐NH_2_ was dissolved in 3 mL of anhydrous methanol in a round‐bottom flask and dried via rotary evaporation to form a thin lipid film. Subsequently, 2 mL of Ru nanozyme aqueous solution (2 mg/mL) was added, and the mixture was subjected to probe ultrasonication for 5 min using a cell disruptor. The resulting product, BP, was collected for subsequent experiments.

### 4.3 Synthesis of BP‐Encapsulated Probiotics (BPEP)

Actively growing *Escherichia coli Nissle 1917* (Probiotics) probiotics were collected and washed twice with sterile PBS (5000 rpm, 5 min, 4°C) to remove residual culture medium. A total of 1 × 10^8^ CFU of probiotics was mixed with 1.15 mg 1‐ethyl‐3‐(3‐dimethylaminopropyl) carbodiimide (EDC) and 1.3 mg N‐hydroxysuccinimide (NHS) and stirred magnetically for 5 min. Subsequently, 1 mL of BP solution (1 mg/mL in sterile PBS) was added, and the mixture was stirred for 3 h at room temperature to allow covalent conjugation. The reaction mixture was then washed three times with sterile PBS (5000 rpm, 5 min, 4°C) to remove unbound BP, and the final BPEP product was resuspended in 1 mL of sterile PBS for further use.

### 4.4 CAT‐like Activity Assay

The CAT‐like activity of Ru nanozyme and BP was evaluated in 15 mL clean centrifuge tubes. In each tube, 2 mL of PBS (pH 6.8) and 40 µL of 0.1 mg/mL Ru nanozyme or BP were mixed. Subsequently, 40 µL of 30% H_2_O_2_ was added to initiate the reaction. The dissolved oxygen concentration was monitored every 1 min using a dissolved oxygen meter for a total of 12 min, and the oxygen generation profile was plotted as a function of time. Within the 2 mL reaction system, the enzyme activity was defined as 1 U when 1 µmol of H_2_O_2_ was decomposed within 1 min. The specific activity was calculated by dividing the measured enzyme activity by the mass of the nanomaterial present in the reaction system, yielding the specific catalase‐like activity in units per milligram (U/mg).

### 4.5 SOD‐Like Activity Assay

The SOD‐like activity of the nanomaterials was measured using the cytochrome c reduction method. In a 300 µL reaction system on a 96‐well plate, 30 µL of deionized water was initially added to each well, followed by 50 µL of xanthine, PBS (pH 6.8), and cytochrome c. The ratio of deionized water to xanthine oxidase was adjusted so that the absorbance change (ΔA_1_) at 550 nm over 1 min stabilized at approximately 0.0225.

Once the blank measurement stabilized, 30 µL of deionized water was replaced with different concentrations of Ru nanozyme, BP, Probiotics, or BPEP, and the absorbance change (ΔA_2_) was recorded. The inhibition rate was calculated using the formula: Inhibition rate (%) = (0.0225−ΔA_2_)/0.0225×100%. The inhibition rate was plotted as a function of nanomaterial concentration to generate a dose–response curve. The half‐maximal inhibitory concentration (IC_50_), corresponding to 50% inhibition, was defined as 1 U of enzyme activity. The specific activity (U/mg) was then calculated based on the IC_50_ value and the mass of nanomaterial in the reaction system.

### 4.6 ABTS Radical Scavenging Assay

ABTS^·^^+^ radicals were generated by mixing equal volumes of 7 nm ABTS aqueous solution and 2.45 mm potassium persulfate, followed by incubation in the dark for 12 h. A clean 96‐well plate was prepared, and 10 µL of each sample (Ru nanozyme and BP) at the same concentration was added to the wells. Each sample was set up with three experimental replicates and three blank controls. Subsequently, 190 µL of ABTS working solution was added to each well. The plate was gently mixed and incubated at room temperature in the dark for 30 min. The absorbance at 734 nm was then measured using a microplate reader. The ABTS radical scavenging rate (%) was calculated as: Scavenging rate (%) = (A_sample_‐A_blank_)/A_control_×100%. Where A_sample_ is the absorbance of the sample with ABTS solution, A_blank_ is the absorbance of solvent (ethanol) without ABTS, and A_control_ is the absorbance of ABTS solution without sample.

### 4.7 DPPH Radical Scavenging Assay

DPPH solution was prepared by dissolving 0.008 g of purple DPPH powder in 100 mL of anhydrous ethanol, mixed thoroughly in the dark. In a 96‐well plate, 5 µL of each sample (Ru nanozyme and BP) was added. For each sample, three wells with an equal volume of ethanol instead of DPPH solution were set as blanks, and three experimental replicates were included. Subsequently, 195 µL of DPPH working solution (or ethanol for blank wells) was quickly added using a multichannel pipette. The plate was incubated at 37°C in the dark for 30 min. The absorbance at 517 nm was measured, and the DPPH radical scavenging rate (%) was calculated as: Scavenging rate (%) = (A_sample_‐A_blank_)/A_control_×100%. Where A_sample_ is the absorbance of the sample with DPPH solution, A_blank_ is the absorbance of ethanol without DPPH, and A_control_ is the absorbance of DPPH solution without a sample.

### 4.8 Cell Culture

The myocardial cells H9c2(2‐1) (RRID: CVCL_0286) were purchased from Wuhan PricellaBiotechnology Co., Ltd. in November 2023. Authentication was conducted by short tandem repeat (STR) profiling at the Institute of Analysis and Testing, Wuhan Procell, confirming a complete match with the reference profile of H9c2 (RRID: CVCL_0286). The human colorectal adenocarcinoma cell line Caco‐2 (RRID: CVCL_0025) was obtained from Servicebio Technology Co., Ltd. (Wuhan, China) in July 2025. Authentication was conducted through short tandem repeat (STR) profiling on January 22, 2024, and the resulting STR profile at the Institute of Analysis and Testing, Wuhan Zishan, confirming a complete accordance with the reference profile of Caco‐2 (RRID: CVCL_0025). Both cell lines were cultured under standard conditions in a humidified incubator at 37°C with 5% CO_2_, using appropriate growth media supplemented with 10% fetal bovine serum and 1% penicillin‐streptomycin.

### 4.9 CCK‐8 Cytotoxicity Assay

The cytotoxicity of BP nanozyme was evaluated using the Cell Counting Kit‐8 (CCK‐8) assay. The H9c2 were trypsinized, centrifuged, and resuspended, then seeded at a density of 5 × 10^3^ cells per well in a 96‐well plate and cultured for 24 h to allow cell attachment and growth. BP nanozyme stock solution was diluted with complete culture medium to prepare seven concentrations: 12.5, 25, 50, 100, 150, 200, 300, and 400 µg/mL. The original medium in each well was carefully removed, and 100 µL of medium containing the corresponding nanozyme concentration was added. Cells were incubated for 24 or 48 h at 37°C in a humidified 5% CO_2_ incubator. Subsequently, 10 µL of CCK‐8 working solution was added to each well, and after incubation according to the manufacturer's instructions, the optical density (OD) was measured using a microplate reader. Cell viability was calculated based on OD values relative to untreated control wells.

### 4.10 Hemolysis Assay

Red blood cells (RBCs) were isolated from mouse blood by centrifugation at 1500 rpm for 10 min and washed twice with PBS. A 0.1 mL aliquot of 5% RBC suspension was resuspended in 2 mL PBS. BP nanozyme at various concentrations (25, 50, 100, 200, and 400 µg/mL, diluted in PBS) was incubated with the RBC suspension at 37°C for 30 min. Deionized water and PBS were used as positive and negative controls, respectively. After incubation, the mixtures were centrifuged, and 100 µL of the supernatant was collected to measure the absorbance at 578 nm using a microplate reader. The hemolysis rate was calculated using the following formula: Hemolysis rate (%) = (OD_sample_‐OD_PBS_)/(OD_ddH2O_‐OD_PBS_) × 100%.

### 4.11 Cell Protection Assay of BP

H9c2 cells were seeded in 96‐well plates at a density of 8 × 10^3^ cells per well and cultured overnight at 37 °C to allow full adhesion. A cellular injury model was established using 500 nM doxorubicin (DOX). BP nanozyme at different concentrations (40, 80, and 120 µg/mL) was added to the cells and co‐incubated for 12 h. Subsequently, 500 nm DOX was added to each well and incubated for an additional 12 h. Cell viability was determined using the CCK‐8 assay according to the manufacturer's instructions, and the absorbance was measured with a microplate reader. The protective effect of BP on H9c2 cells was evaluated by comparing cell viability across different treatment groups.

### 4.12 Live/Dead Cell Staining Assay

The protective effect of BP nanozyme on H9c2 cells under DOX–induced injury was evaluated using a Calcein‐AM/PI Cell Viability/Cytotoxicity Assay Kit. Three experimental groups were set up: (1) Control; (2) DOX injury group; (3) BP + DOX group. H9c2 cells were harvested, centrifuged, and resuspended in complete medium at a density of 1.0 × 10^5^ cells/mL. Cells were seeded in six‐well plates at 2 mL per well and cultured for 24 h. BP was diluted in complete medium to the desired concentrations. The original medium was removed, and each well was washed once with 1 mL PBS. Control and DOX groups were supplemented with 2 mL drug‐free complete medium, whereas the BP + DOX group received 2 mL complete medium containing the corresponding concentration of BP. Cells were incubated for 12 h. After incubation, the medium was removed, and the cells were washed twice with PBS. Except for the Control group, the other two groups were treated with 2 mL of complete medium containing 500 nm DOX and incubated for 6 h.

After incubation, the medium was removed, and the cells were washed twice with PBS. Each well was then treated with 1 mL of Calcein‐AM/PI staining solution and incubated at 37°C in the dark for 30 min. Fluorescence images were acquired using an inverted fluorescence microscope to assess cell viability and cytotoxicity.

### 4.13 Mitochondrial Membrane Potential Assay

H9c2 cells were seeded in six‐well plates at a density of 2.5 × 10^5^ cells per well and incubated at 37°C overnight to allow full attachment. Cells were then co‐incubated with 50 µg/mL BP for 12 h. Except for the control group, all wells were treated with 500 nm DOX for 6 h. JC‐1 staining solution was freshly prepared by mixing 25 µL of 200× JC‐1 dye with 4 mL ultrapure water and vortexing thoroughly to ensure complete dissolution. Subsequently, 1 mL of 5× JC‐1 staining buffer was added and immediately mixed. The old medium was removed, and cells were washed three times with PBS. 1 mL of the freshly prepared JC‐1 staining solution was carefully added along the wall of each well. Cells were incubated at 37°C for 20 min. After incubation, the staining solution was removed, and cells were washed twice with 1× JC‐1 staining buffer. Finally, 2 mL of fresh culture medium was added per well, and fluorescence images were acquired using a fluorescence microscope to evaluate mitochondrial membrane potential.

### 4.14 Cell Apoptosis Assay

The protective effect of BP nanozymes on H9c2 cells under DOX‐induced injury was evaluated using a Calcein‐PI cell viability/cytotoxicity detection kit. Three experimental groups were established: (1) Control, (2) DOX injury, and (3) BP + DOX. H9c2 cells were trypsinized, centrifuged, and resuspended in complete medium at a density of 1.0 × 10^5^ cells/mL. Subsequently, 2 mL of cell suspension was seeded into each well of a six‐well plate and incubated for 24 h. BP nanozyme was diluted in complete medium, and the original medium in each well was carefully removed. Cells were washed once with 1 mL PBS. Control and DOX groups received 2 mL fresh complete medium, while the BP group received 2 mL complete medium containing the corresponding concentration of BP, followed by 12 h incubation. Afterward, the medium was removed, and the cells were washed twice with PBS. Except for the control group, the DOX and BP groups were treated with 2 mL of 500 nM DOX diluted in complete medium and incubated for 12 h. Cells were then washed once with PBS and digested using EDTA‐free trypsin. The cell suspension was centrifuged at 1800 rpm for 5 min at 4°C, and the supernatant was discarded. Cells were washed twice with pre‐cooled PBS under the same centrifugation conditions and resuspended in 100 µL 1× Binding Buffer to obtain a single‐cell suspension. For apoptosis staining, 5 µL Annexin V‐FITC and 5 µL PI staining solution were added to each sample, gently mixed, and incubated at room temperature in the dark for 10 min. Subsequently, 400 µL of 1× Binding Buffer was added and mixed gently. Flow cytometry analysis was performed within 1 h to detect apoptotic cell populations.

### 4.15 Intracellular ROS Evaluation Using DCFH‐DA Fluorescent Probe

The intracellular reactive oxygen species (ROS) scavenging effect of BP nanozymes in H9c2 cells under DOX‐induced injury was assessed using a ROS detection kit and flow cytometry. H9c2 cells were seeded onto 12‐well plates at a density of 1 × 10^5^ cells/well, with cell climbing slices pre‐placed at the bottom, and incubated overnight at 37°C to allow attachment. Three experimental groups were established: Control, DOX, and BP. After adherence, the medium in all wells was removed. The BP group was treated with BP (50 µg/mL, diluted in complete medium) and incubated at 37°C for 12 h. Subsequently, 500 nm DOX was added to the DOX and BP groups and co‐incubated for 6 h. Cells were washed three times with sterile PBS to remove residual medium. For ROS detection, the DCFH‐DA probe was diluted 1:1000 in serum‐free medium to a final concentration of 5µ, with a total volume of 5 mL. The probe solution (1 mL per well) was gently added along the well wall and incubated at 37°C for 30 min in the dark. During incubation, the plates were gently tilted every 4–5 min to ensure sufficient contact between the probe and cells. After staining, the probe solution was removed, and cells were washed three times with PBS to remove excess probe. Fluorescence images were acquired using a laser confocal microscope (excitation: 488 nm; emission: 520 nm), and flow cytometry was employed to quantitatively evaluate intracellular ROS levels.

### 4.16 Evaluation of Cellular Uptake in Caco‐2 Cells

The cellular uptake of Ru nanozyme was evaluated by incubating Caco‐2 cells with the nanostructures for 6 h. After incubation and subsequent washing steps, the cells were subjected to hyperspectral microscopy analysis to determine the fluorescence intensity and distribution profile of the internalized nanozymes.

### 4.17 Animals

Male Balb/C mice (6 weeks old) were purchased from Jicui Yaokang (China) and acclimated for 1 week under specific pathogen‐free (SPF) conditions before experiments. All animal experiments were conducted in accordance with the protocol approved by the Animal Ethics Committee of the Experimental Animal Center of Zhengzhou University (No. ZZU‐LAC20240322[20]). All procedures followed the guidelines outlined in the “Guide for the Care and Use of Laboratory Animals”.

### 4.18 Statistical Analysis

All experimental data were analyzed using GraphPad Prism 8.0.2 software and are presented as mean ± standard deviation (Mean ± SD). For comparisons between multiple groups, one‐way analysis of variance (ANOVA) followed by Tukey's multiple comparisons test or Dunnett's multiple comparisons test was applied as appropriate. For datasets involving two independent variables, two‐way ANOVA with Sidak's multiple comparisons test was used. Statistical significance was denoted as *p* < 0.01, *p* < 0.005, *p* < 0.001, *p* < 0.0001, and “ns” indicates no significant difference.

## Conflicts of Interest

The authors declare no conflicts of interest.

## Supporting information




**Supporting File**: advs75199‐sup‐0001‐SuppMat.docx.

## Data Availability

The data that support the findings of this study are available from the corresponding author upon reasonable request.
