# Supplementary material for: Oral Bioinspired Peroxisome‐Engineered Probiotics for Modulating Gut Microbiota Homeostasis and Alleviating Cardiac Chemotherapy Toxicity
Source: Adv Sci (Weinh). 2026 May 8:e19344. Online ahead of print. doi: 10.1002/advs.202519344 (PMC13334650; doi:10.1002/advs.202519344)
Supplement: Supplementary file 1 — Supporting File: advs75199‐sup‐0001‐SuppMat.docx. [file ADVS-9999-e19344-s001.docx]

Supporting Information

**Oral Bioinspired Peroxisome-Engineered Probiotics for Modulating Gut Microbiota Homeostasis and Alleviating Cardiac Chemotherapy Toxicity**

Shuyu Wang^1,2, #^, Xiaowan Fan^5,6, #^, Chao Zhang^2^, Shuai Han^6^, Yike Guo^4^, Yuchen Wang^4^, Wei Wang^6^, Ying Liu^2*^, Wei Jiang^2,3,4*^, Xiaoying Chen^1*^

^1^Cardiovascular Institute of Zhengzhou University, Department of Cardiology, The First Affiliated Hospital of Zhengzhou University, Zhengzhou, China.

^2^National Health Commission Key Laboratory of Cardiovascular Regenerative Medicine, Central China Subcenter of National Center for Cardiovascular Diseases, Henan Cardiovascular Disease Center, Fuwai Central-China Cardiovascular Hospital, Central China Fuwai Hospital of Zhengzhou University, Zhengzhou 450046, China.

^3^Institute of Cardiovascular Disease, Henan Academy of Innovations in Medical Science, Zhengzhou, Henan 451162, China.

^4^State Key Laboratory of Metabolic Dysregulation & Prevention and Treatment of Esophageal Cancer, Tianjian Laboratory of Advanced Biomedical Sciences, Academy of Medical Sciences, Zhengzhou University, Zhengzhou 450052, China.

^5^School of Medicine of Henan University, Zhengzhou 450046, Henan, China.

^6^ Nanozyme Laboratory in Zhongyuan, Henan Academy of Innovations in Medical Science, Zhengzhou, Henan 451163, China.

#These authors contributed equally to this work.

*Corresponding Authors: [fwhzliuying@zzu.edu.cn](mailto:fwhzliuying@zzu.edu.cn) (Y. Liu); [weijiang@zzu.edu.cn](mailto:weijiang@zzu.edu.cn) (W. Jiang); [Chenxiaoying_mail@163.com](mailto:Chenxiaoying_mail@163.com) (X. Chen)

**Keywords:** doxorubicin-induced cardiotoxicity, gut-heart axis, nanozyme, probiotics, oral targeted therapy.

**Supplementary Figures**

**
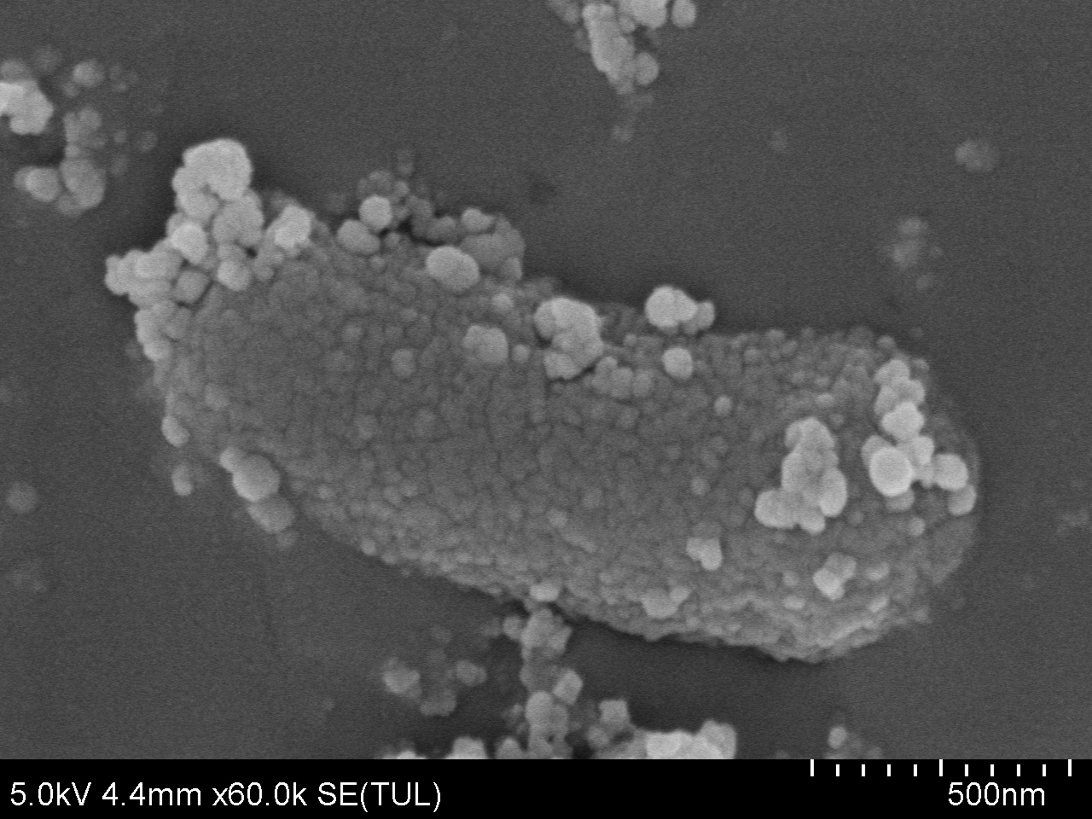
**

Figure S1. SEM image of BPEP, scale bar =500 nm.

**
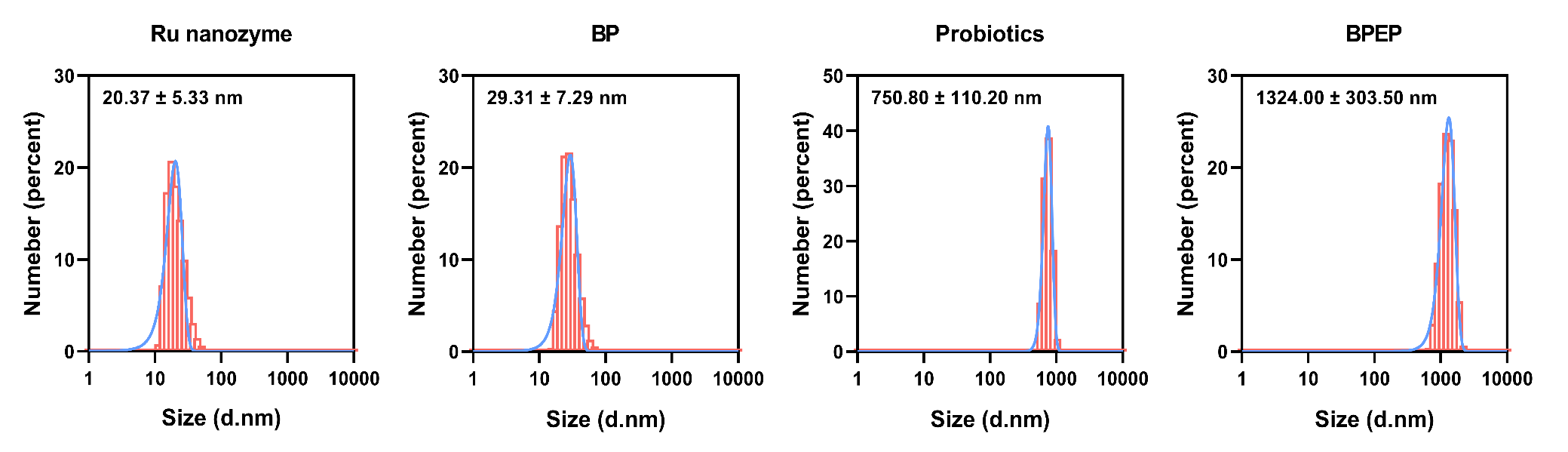
**

Figure S2. Hydrodynamic diameters of Ru nanozyme, BP, Probiotics, and BPEP nanozymes.

**
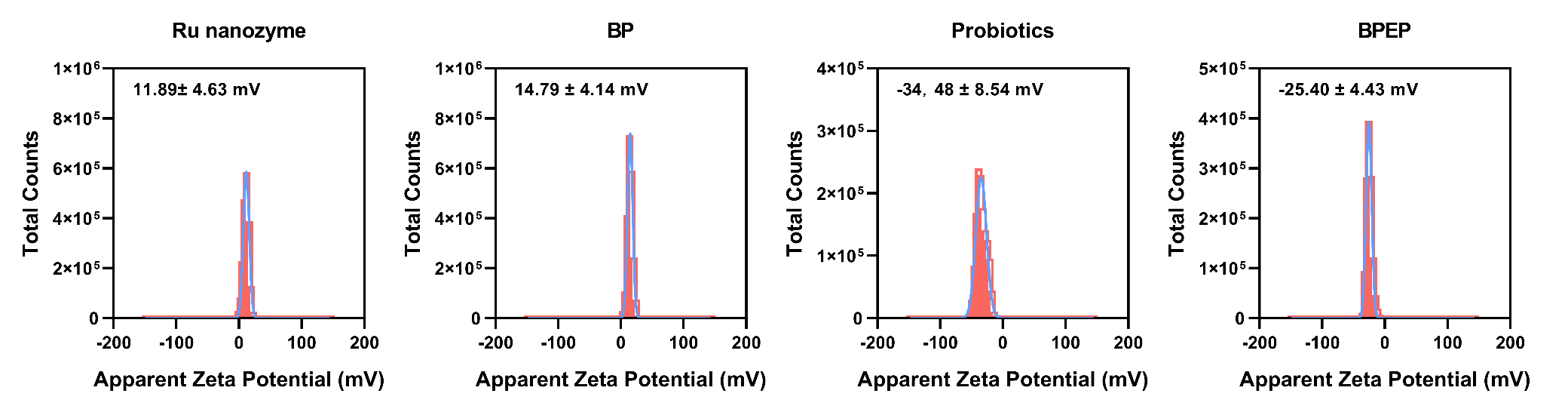
Figure S3**. Zeta potentials of Ru nanozyme, BP, Probiotics, and BPEP nanozymes.


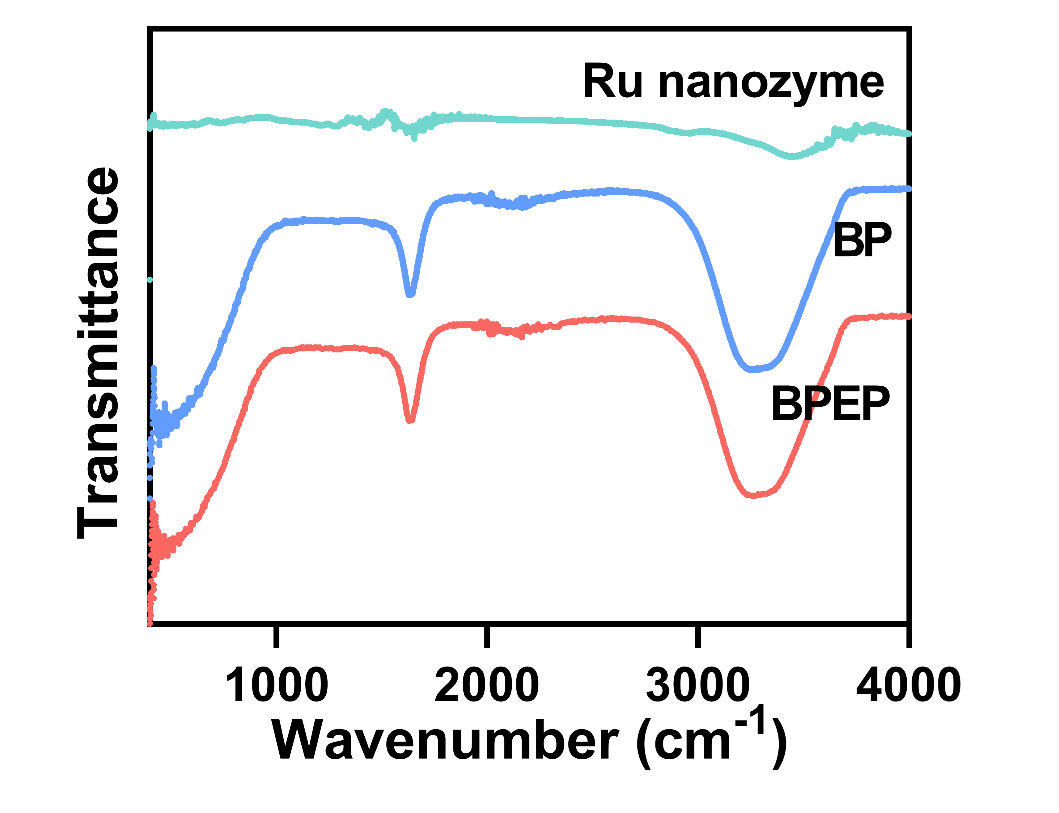


Figure S4. FTIR spectra of Ru nanozyme, BP, and BPEP nanozymes.


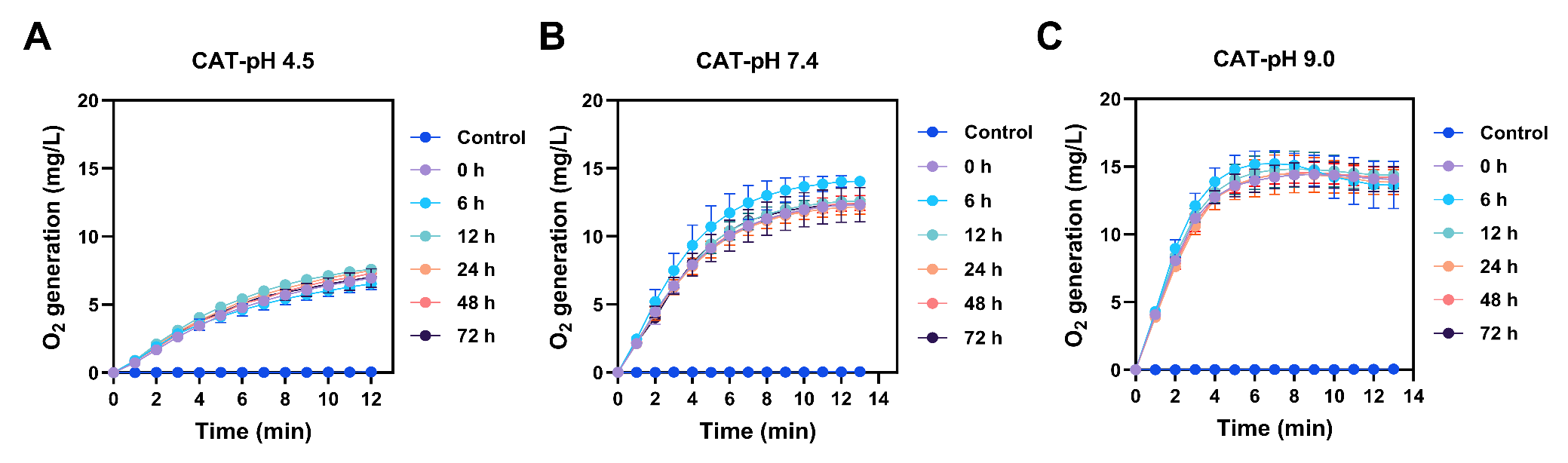


Figure S5. CAT-like activity of BP measured at different time points under varying pH conditions: (A) pH 4.5, (B) pH 7.4, and (C) pH 9.0. (n = 3).


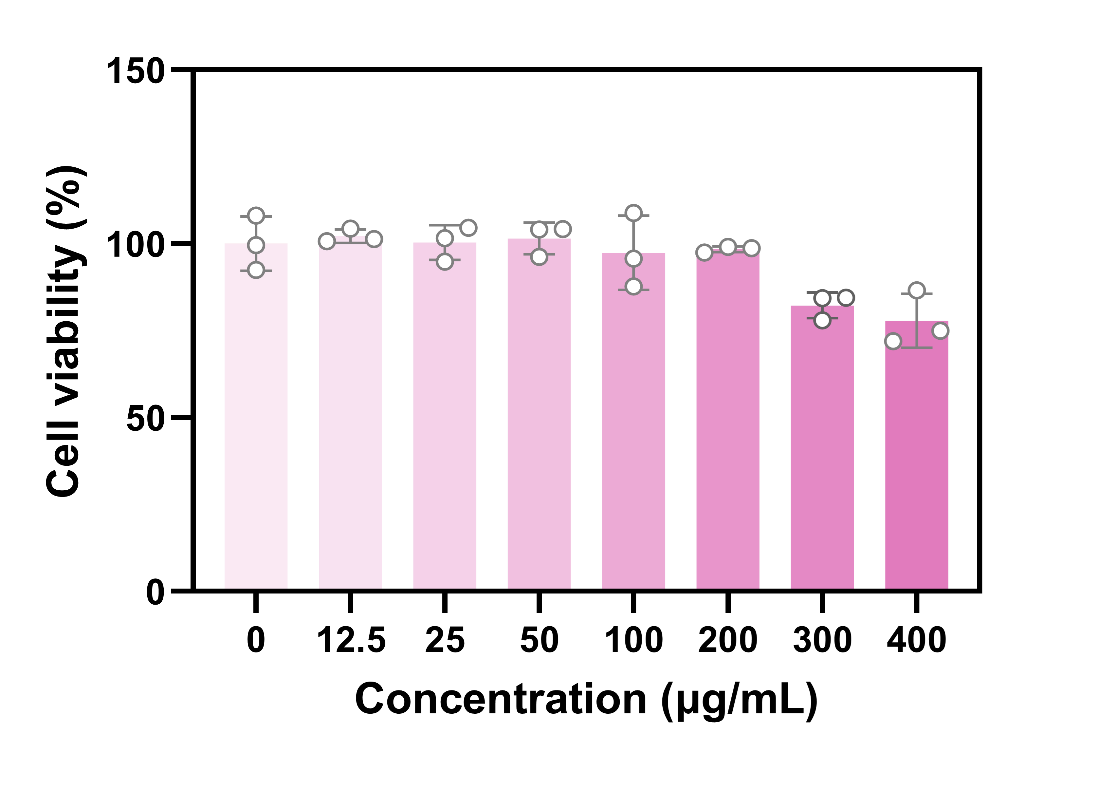


Figure S6. Cell viability of H9c2 cells after 48 h exposure to different concentrations of BP (n = 3).


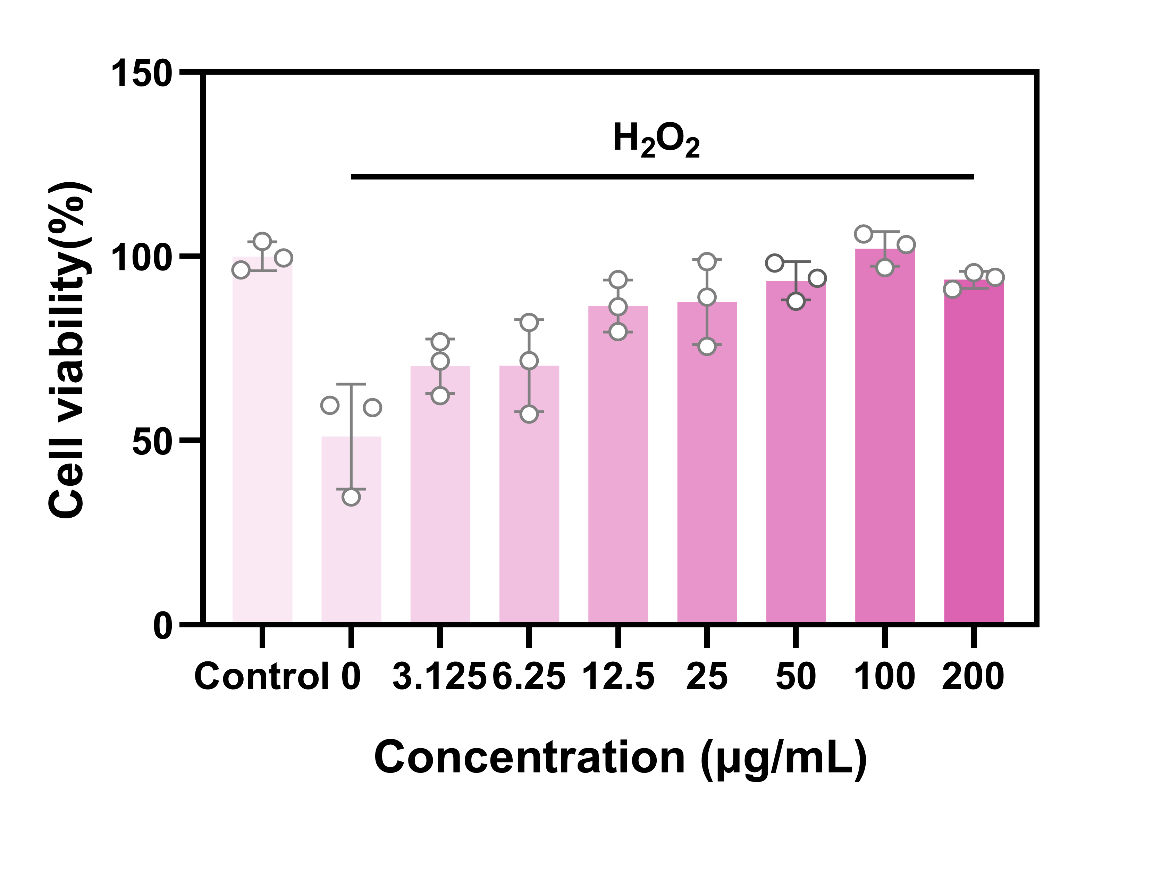


Figure S7. Protective effects of BP at varying concentrations on H9c2 cells under 200 μm H_2_O_2_-induced injury (n = 3).


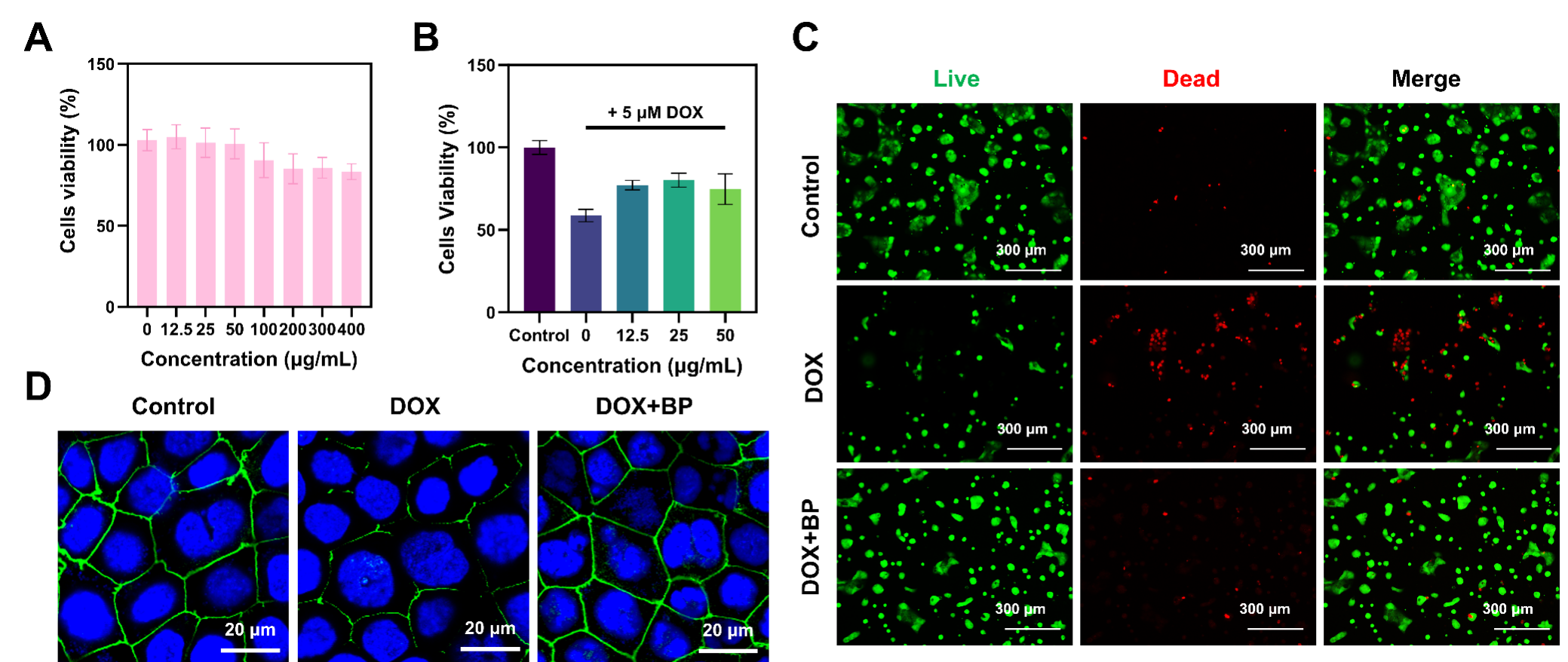


**Figure S8.** *In Vitro* Protective Effects of BP on DOX-Induced Intestinal Epithelial Injury. (A) Cell viability of Caco-2 cells after treatment with different concentrations of BP. (B) Protective effect of BP at various concentrations against DOX (5 μM)-induced reduction in Caco-2 cell viability. (C) Representative live/dead staining images of Caco-2 cells under different treatments，scale bar = 20 μm. (D) Representative immunofluorescence images showing ZO-1 expression, illustrating that BP alleviates DOX (5 μM)-induced tight junction disruption in Caco-2 cells, scale bar = 300 μm.


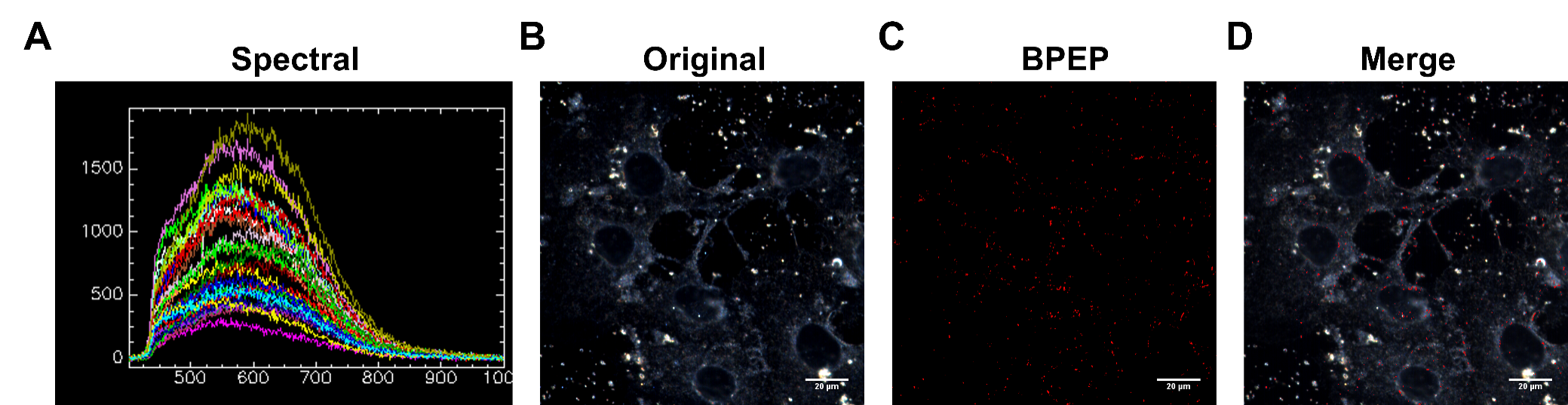
Figure S9. Hyperspectral microscopy analysis of cellular uptake. (A) Spectral library of BPEP. (B) Dark-field image of cells. (C) Characteristic spectral profile of BPEP used for mapping its distribution. (D) Confocal images of Caco-2 cells incubated with BPEP.


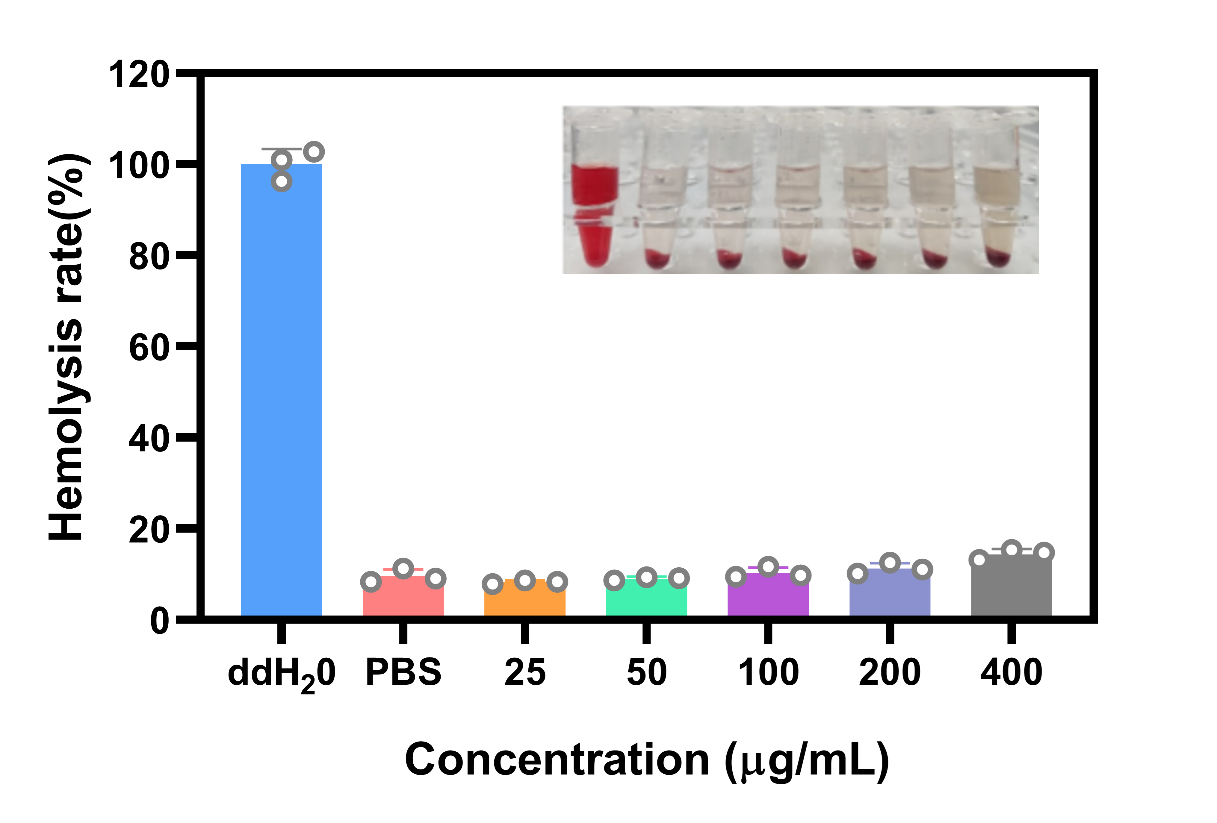


Figure S10. Hemocompatibility assessment of BP *via* hemolysis assay (n=3).


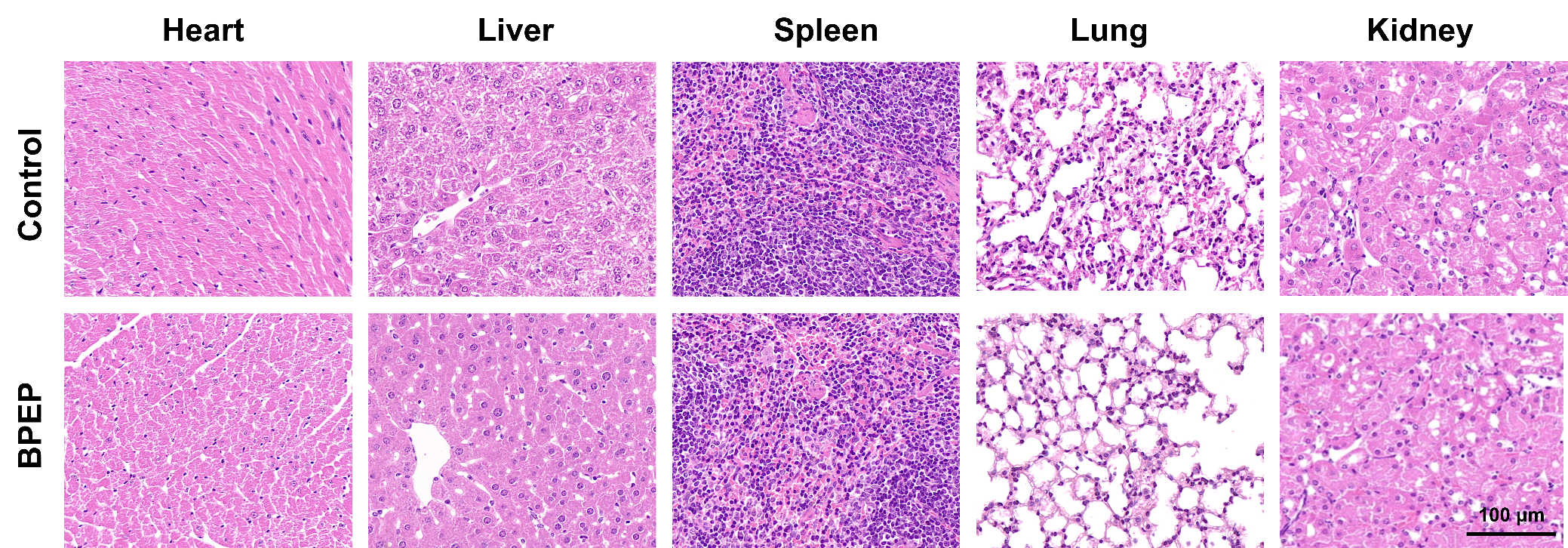
Figure S11. H&E staining images of major organs from mice in different treatment groups. Scale bar = 100 μm.


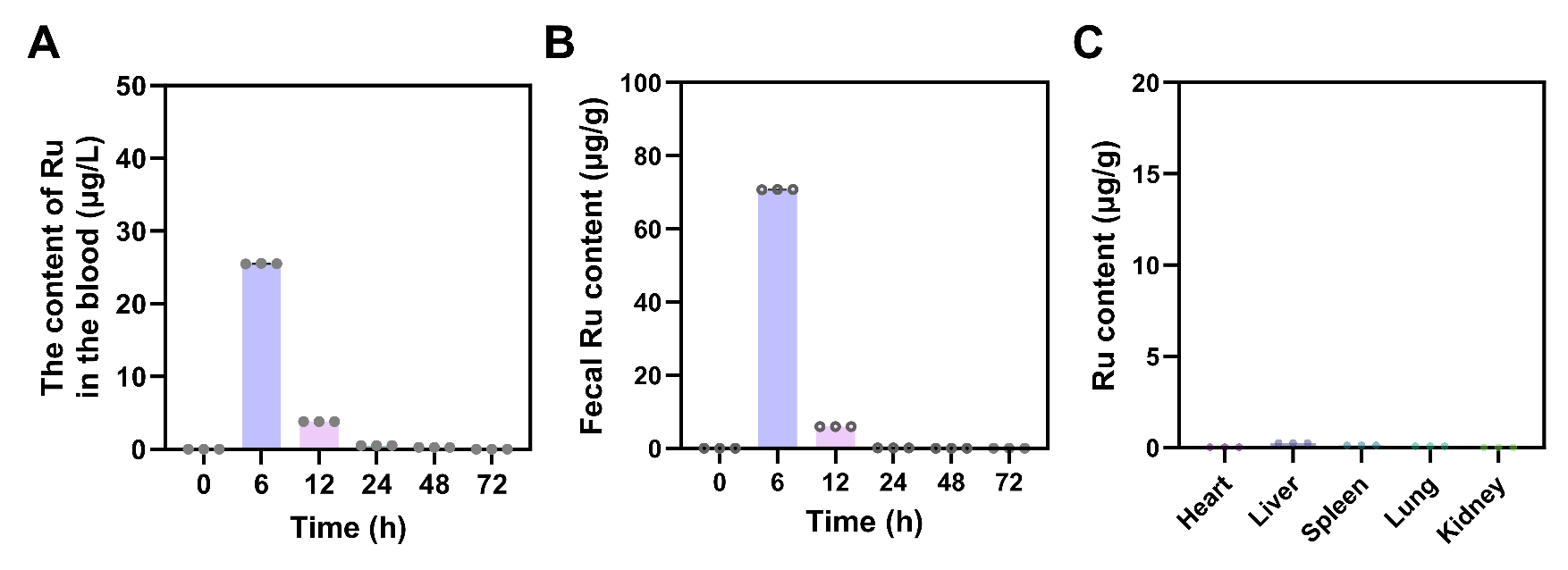


**Figure S12*.*** *In vivo* Ru clearance from BPEP measured by ICP-MS. (A) Time-dependent Ru levels in blood after oral administration (n = 3). (B) Ru content in feces at corresponding time points (n = 3). (C) Ru levels in major organs (heart, liver, spleen, lungs, kidneys) at 72 h (n = 3).


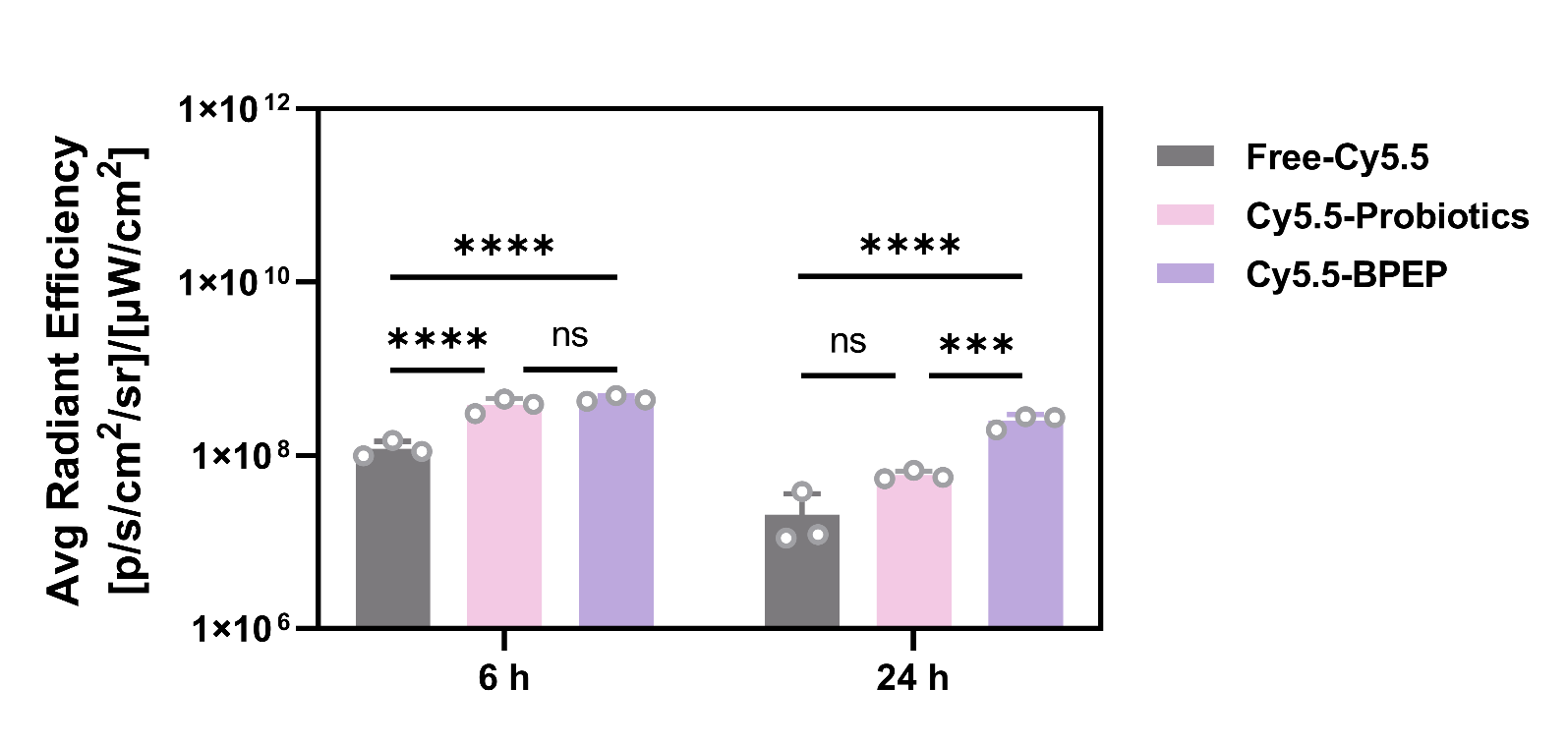


Figure S13. *Ex vivo* fluorescence intensity analysis of isolated intestines collected at 6 and 24 h after oral administration of Free-Cy5.5, Cy5.5-Probiotics, or Cy5.5-BPEP (n = 3; two-way ANOVA with Sidak’s multiple comparisons test; ****p* < 0.001, *****p* < 0.0001).

**
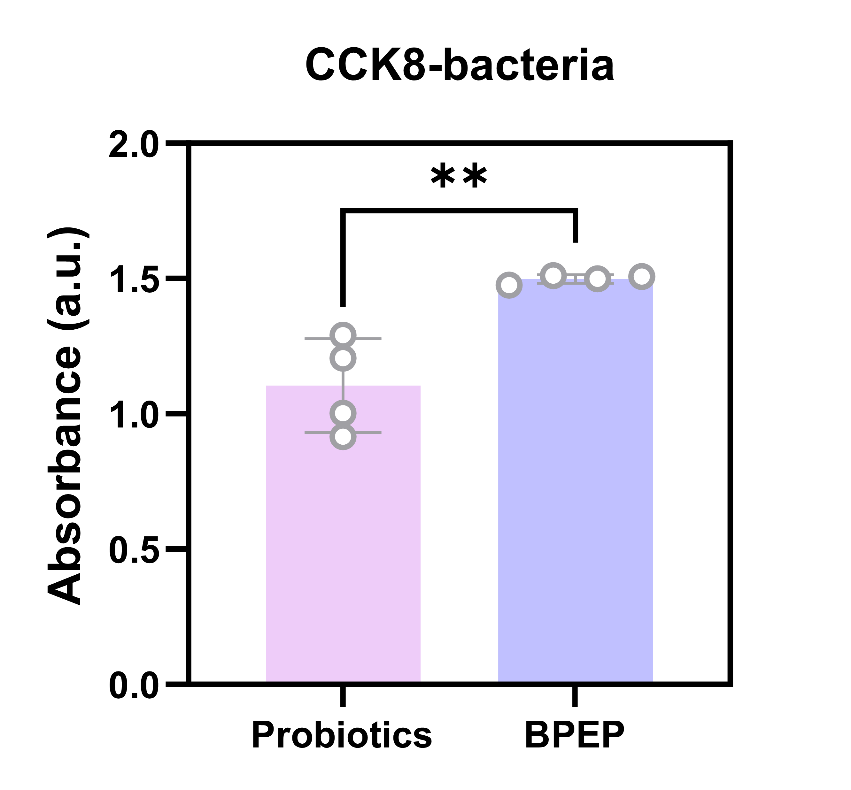
**

**Figure S14**. CCK-8 analysis of gut contents 24 h after oral administration of Probiotics or BPEP shows higher bacterial viability in the BPEP group (n = 3; unpaired two-tailed Student’s t-test, ***p* < 0.01).


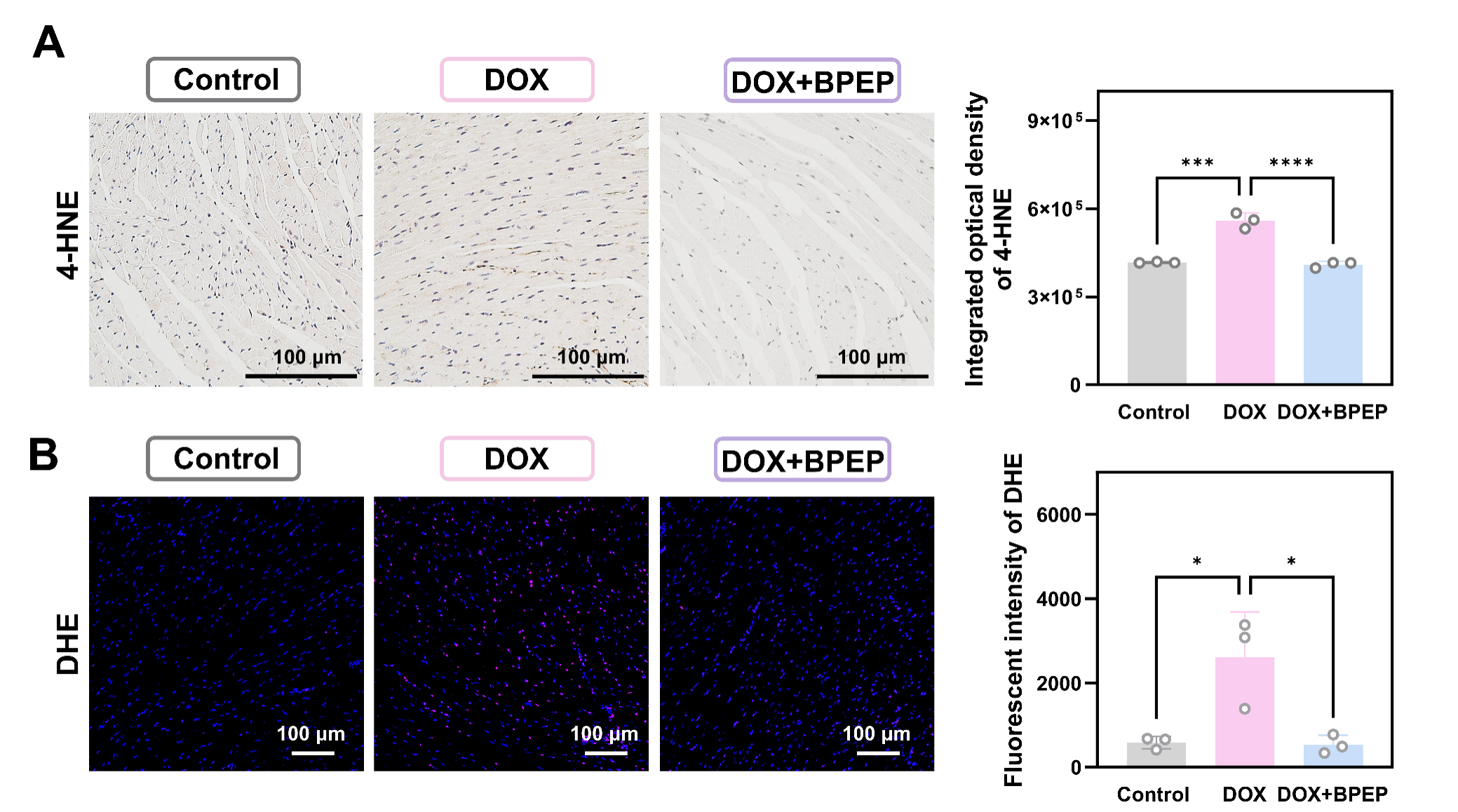


Figure S15. BPEP attenuates DOX-induced cardiac oxidative stress. (A) Representative immunohistochemical images of 4-HNE in heart tissue sections from different treatment groups, with quantitative analysis (n = 3; one-way ANOVA with Tukey’s multiple comparisons test; ****p* < 0.001). (B) Representative DHE fluorescence images indicating ROS levels in heart tissue sections from each group, with quantitative analysis (n = 3; one-way ANOVA with Tukey’s multiple comparisons test; **p* < 0.05).


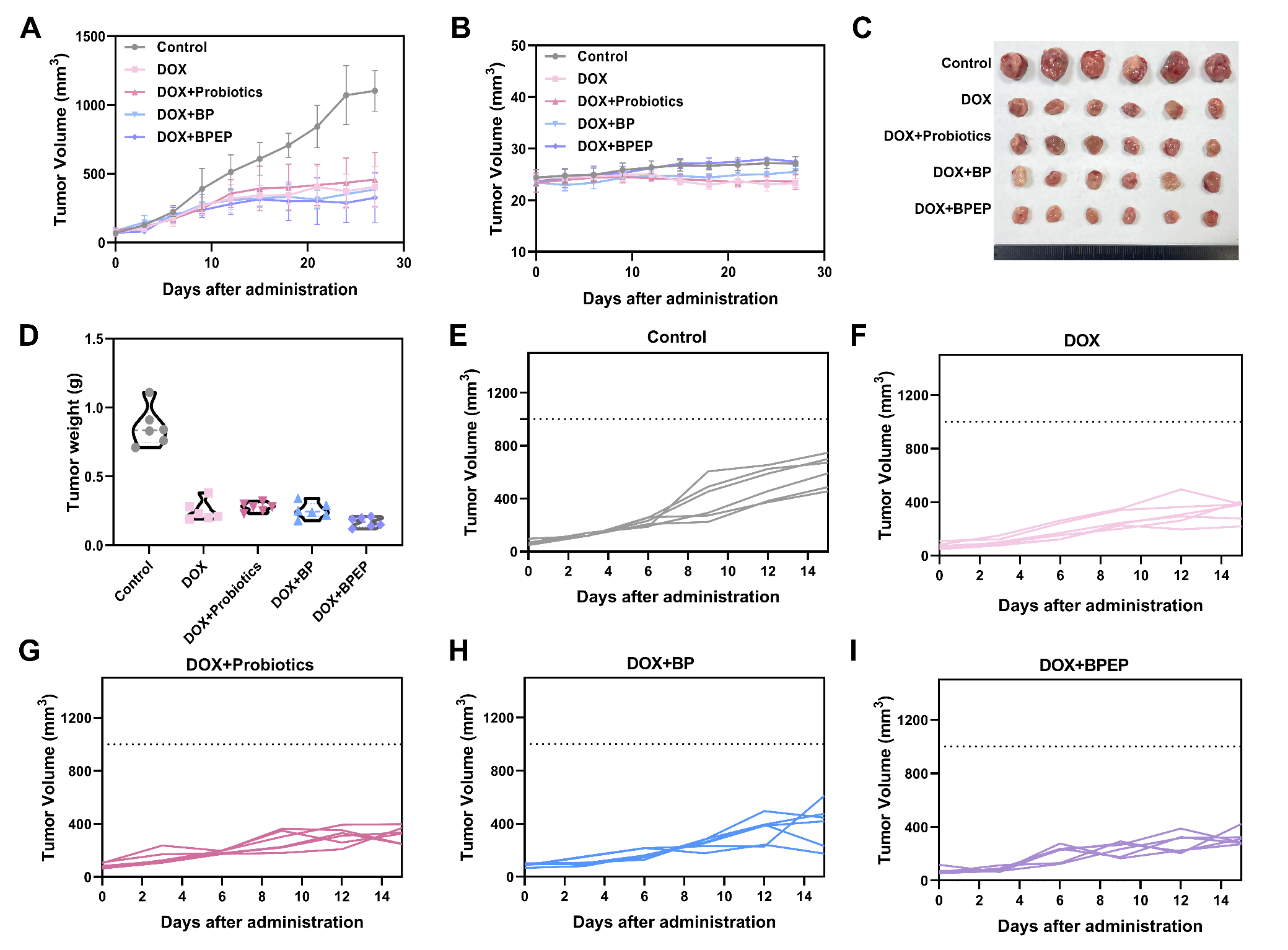


Figure S16. Anti-tumor efficacy evaluation of different treatment groups *in vivo*. (A) Tumor growth curves of mice in different treatment groups (n = 6). (B) Body weight changes during treatment (n = 6). (C) Representative *ex vivo* tumor images from different treatment groups (n = 6). (D) Tumor weights of mice in different treatment groups (n = 6). (E-I) Individual tumor growth curves of mice in each treatment group (n = 6).


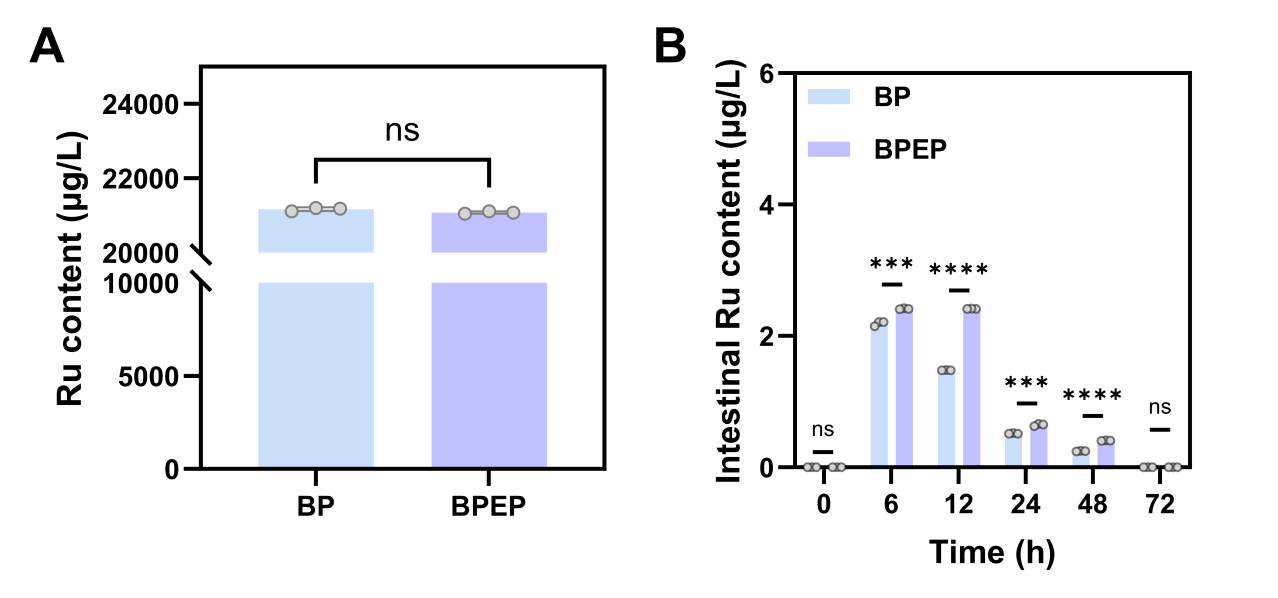


Figure S17. Intestinal retention and Ru quantification of BP and BPEP nanozymes. (A) Ru content of BP and BPEP formulations measured by ICP‑MS prior to administration (n = 3; unpaired two-tailed Student’s t-test). (B) Intestinal Ru levels at 0, 6, 12, 24, 48, and 72 h after oral administration of BP or BPEP, measured by ICP‑MS (n = 3).

**
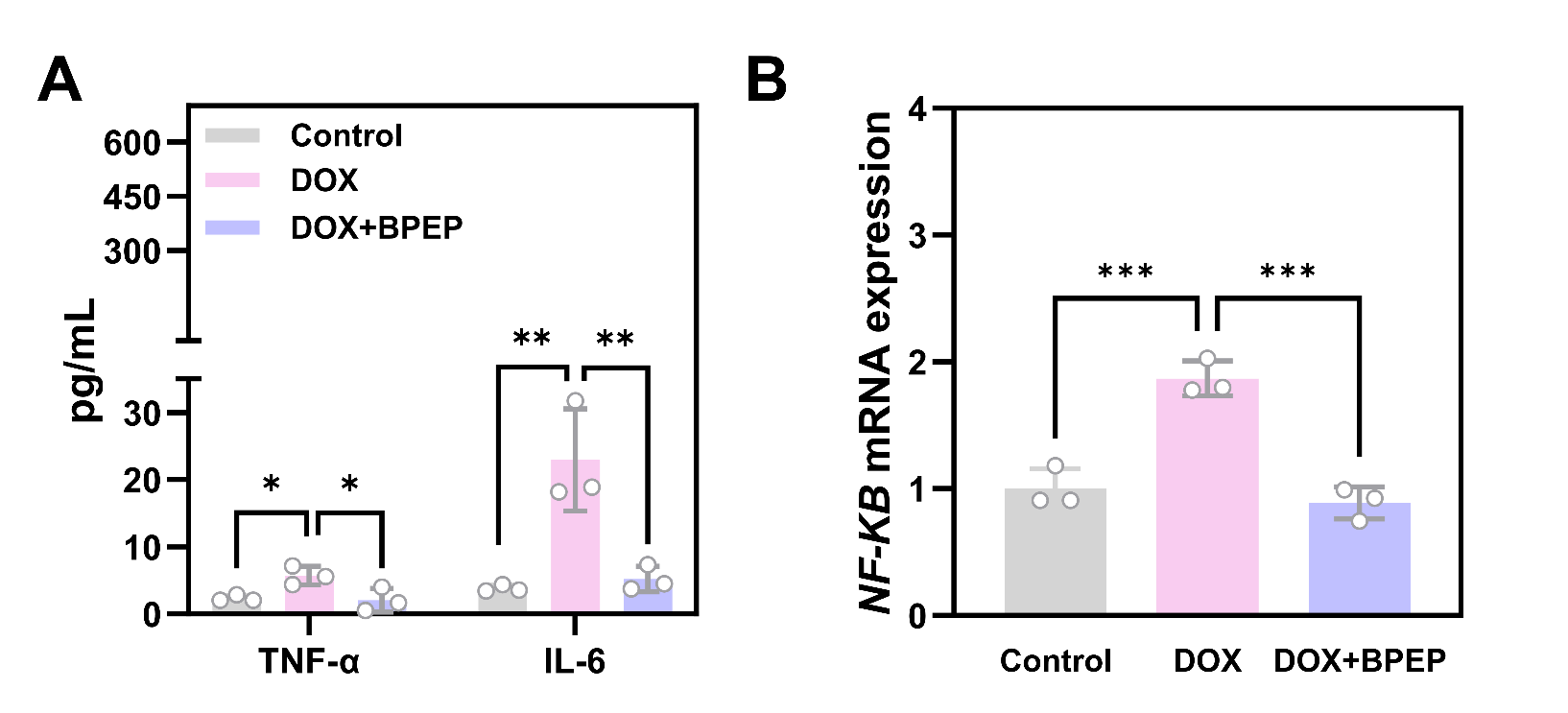
**

**Figure S18.** BPEP reduces systemic inflammation and cardiac NF-κB expression. (A)Serum TNF-α and IL-6 levels in indicated groups (n = 3; one-way ANOVA with Tukey’s multiple comparisons test; **p* < 0.05, ***p* < 0.01). (B) Relative mRNA expression of NF-κB in cardiac tissue (n = 3; one-way ANOVA with Tukey’s multiple comparisons test; ****p* < 0.001).


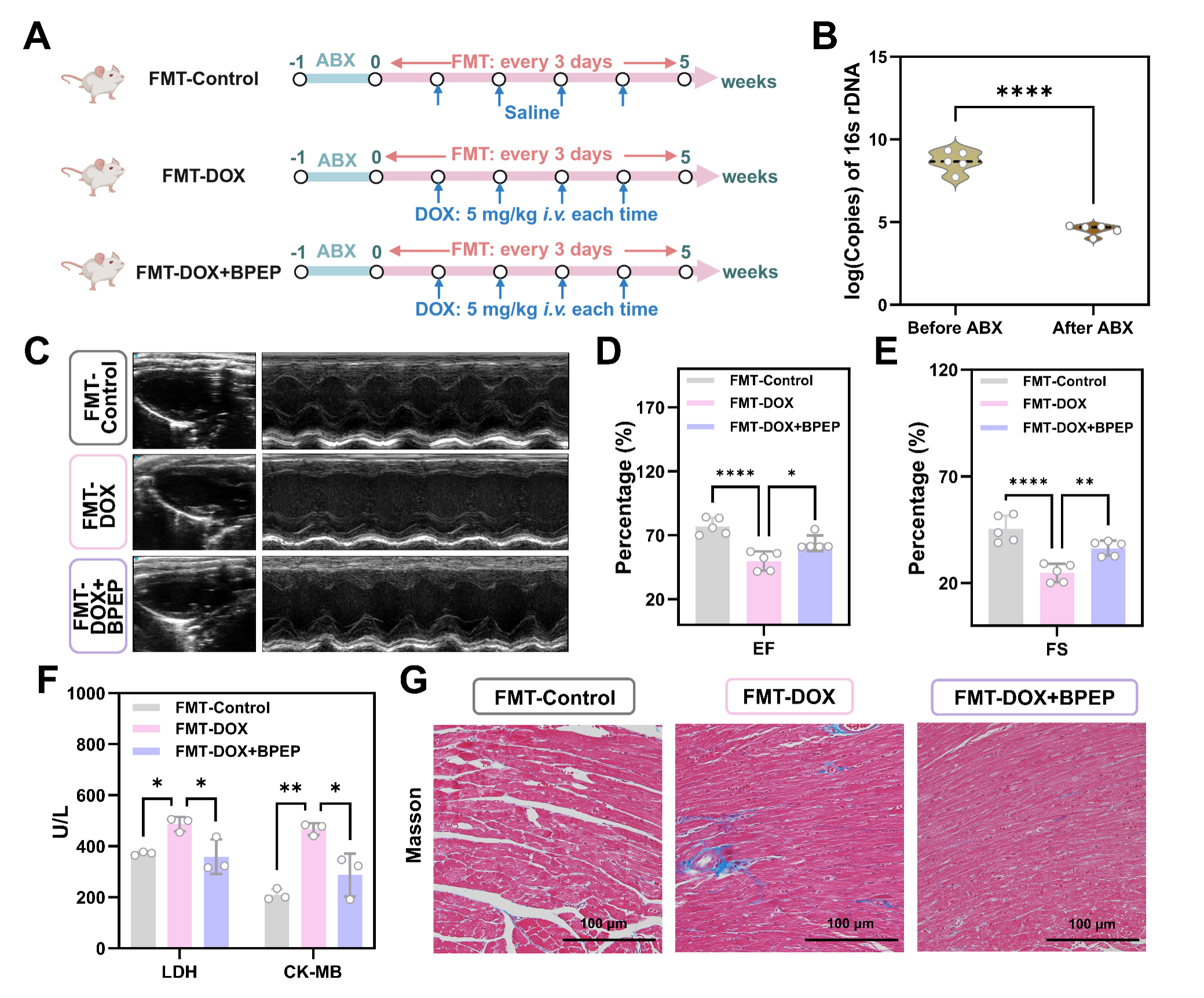


Figure S19. Fecal microbiota transplantation (FMT)-mediated validation of microbiota-dependent cardioprotection. (A) Schematic illustration of the FMT experimental design. (B) Bacterial 16S rDNA copy numbers detected in fecal samples before and after antibiotic (ABX) treatment (n = 5; unpaired two-tailed Student’s t-test, *****p* < 0.0001). (C) Representative echocardiographic images of mouse hearts from different treatment groups at the end of the experimental period. (D, E) Quantification of ejection fraction (EF%) (D) and left ventricular fractional shortening (FS%) (E) in each group (n = 5; one-way ANOVA with Tukey’s multiple comparisons test; **p* < 0.05, ***p* < 0.01, *****p* < 0.0001). (F) Serum levels of LDH and CK-MB in different groups (n = 3; two-way ANOVA with Sidak’s multiple comparisons test; **p* < 0.05, ***p* < 0.01, *****p* < 0.0001). (G) Representative Masson’s trichrome staining of cardiac tissues from different treatment groups. Scale bar = 100 μm.
